# Assessment of the contribution of utility vault water to surface water pollution

**DOI:** 10.1007/s10661-019-7585-y

**Published:** 2019-06-26

**Authors:** Jeremy T. Laurin, Allison C. Luengen

**Affiliations:** 1 Environmental Sciences Department, University of San Francisco, 2130 Fulton Street, San Francisco, CA 94117 USA; 2 Environmental Management, Pacific Gas & Electric Company, 3401 Crow Canyon Road, San Ramon, CA 94583 USA

**Keywords:** Utility vault water, Copper and zinc runoff, San Francisco Bay Pollution, Stormwater management, Urban non-point source pollution, National pollutant discharge elimination system (NPDES) regulations

## Abstract

Utility vaults and underground structures house essential telecommunications, gas, and electrical infrastructure (e.g., transformers, copper wiring) that could contaminate water which accumulates in them. Water is removed from utility vaults during routine infrastructure maintenance. That water is typically released to the storm drain system, raising concerns that polluted water could reach receiving waters. However, no one has measured pollutants in utility vault water. The State Water Resources Control Board (SWRCB) has mandated such measurements as a condition of renewing the National Pollutant Discharge Elimination System Utility Vault Permit. We analyzed 126 priority pollutants in 20 utility vault water samples collected throughout California by Pacific Gas and Electric Company (PG&E). We also estimated the volume of utility vault water discharged and calculated loads. Twenty-one priority pollutants were detected. Metals were commonly found. Only copper (Cu) and zinc (Zn) exceeded water quality criteria. Their maximum concentrations were 791 and 386 μg/L, respectively. Median Cu and Zn concentrations of 9.66 and 81.6 μg/L were representative of urban stormwater, suggesting runoff is a source of metals in utility vault water. For San Francisco Bay, Cu and Zn loads from PG&E's utility vault water (0.06 and 0.5 kg/year) were inconsequential compared to previously reported total loads (74,000 and 320,000 kg/year) from stormwater, wastewater treatment plants, etc. For California, utility vault water loads were 5 and 40 kg/year of Cu and Zn. We are the first to report pollutant concentrations in utility vault water. Utility vaults are not a major source of pollutants to receiving waters.

**Electronic supplementary material:**

The online version of this article (10.1007/s10661-019-7585-y) contains supplementary material, which is available to authorized users.

## Introduction

In the USA, the Clean Water Act of 1972 successfully addressed many point sources of pollution, providing funding and reducing pollutant loads into surface waters (Sanudo-Wilhelmy et al. 2004). However, the Clean Water Act does not effectively control the more difficult to manage non-point pollution sources (Dressing et al. 2016; Lyon and Stein 2009; U.S. Environmental Protection Agency 1996; Wright and Welbourn 2002). Non-point source pollution is now one of the major global threats to water quality, especially in urban areas where stormwater run-off can mobilize pollutants (Fraga et al. 2016; Gilbreath and McKee 2015). Consistent with this focus, diffuse sources of pollution have come under scrutiny. For example, water accumulated within underground utility vaults has not previously been studied to determine its source or impact on receiving waters.

**Fig. 1:**
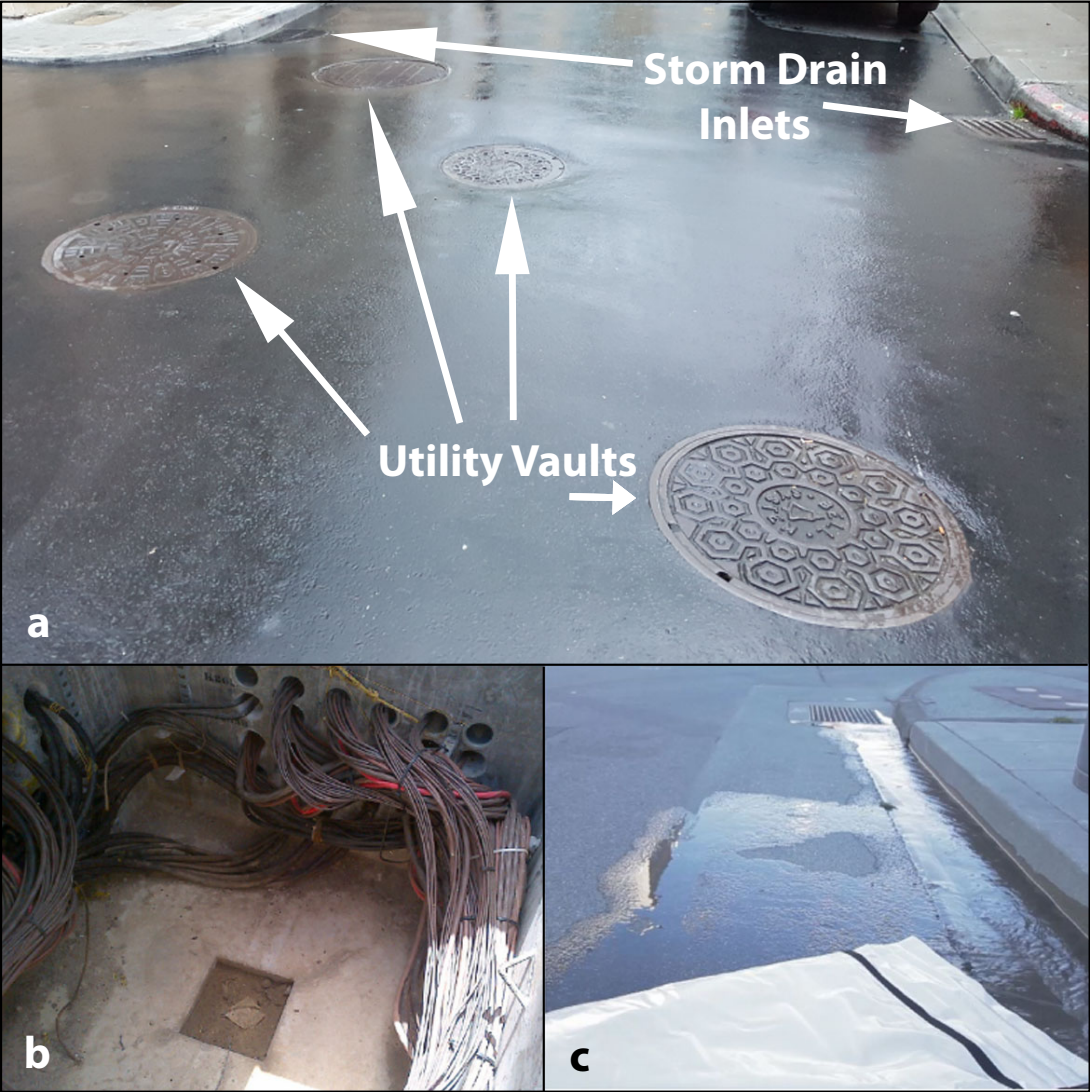
**a** Utility vaults are often found in close proximity to storm drain inlets in urban areas, including this photo from downtown San Francisco, California. **b** This PG&E utility vault from Morrow Bay, California houses only wires (i.e., pull/switch box type) and is currently dry. **c** Water discharged from a utility vault is being passed through a filter sock (in foreground), as per PG&E standard practice (Pacific Gas and Electric Company 2015a, b, c, d, e)

Utility service companies install and maintain sub-surface structures, called utility vaults (Fig. 1). These structures hold equipment used by gas and electrical industries as well as phone, cable, and internet service providers (California State Water Resources Control Board 2014a). Water can enter utility vaults from a variety of sources including stormwater runoff (thought to be the main source), groundwater intrusion, and/or tidal in-flow (California State Water Resources Control Board 2014a). The accumulated water must then be removed (i.e., dewatering) to safely access the equipment (California State Water Resources Control Board 2014a).

While non-point source pollution originates throughout the urban landscape, at its discharge point, it can be regulated as a point source. For example, water found in underground utility vaults is a regulated point source release under the National Pollution Discharge Elimination System (NPDES) program administered by the California State Water Resources Control Board (SWRCB). The trigger for regulation is due to the potential addition of contamination by equipment housed within utility vaults into California surface waters, even though utility vault water consists of non-point source inputs. Within the most recent iteration of the Utility Vault Discharge NPDES Permit (2014), the SWRCB expressed concern that contact with utility infrastructure could release pollutants including oil and grease, petroleum hydrocarbons, and total suspended solids. Oil pollution potential exists within utility vaults because oil is used as an insulating agent within equipment such as transformers, cables, connectors, and wires (California State Water Resources Control Board 2014a).

Although the SWRCB does not call-out additional pollutants as being of particular concern within the 2014 NPDES Utility Vault Permit, it does require permittees to identify likely pollutants. In pollution prevention plans filed with the SWRCB, potential pollutants called out by major utilities frequently included oil and grease, sewage, litter, suspended solids, petroleum hydrocarbons, copper, lead, zinc, and PCBs (Pacific Gas and Electric Company 2015a, b, c, d, e; Sacramento Municipal Utility District 2015; Southern California Edison 2015). For example, Pacific Gas and Electric Company (PG&E), the largest utility company in California, describes the potential for copper and zinc pollution: “Copper may be present due to a core of Cu contained in most cables that may be released into utility vaults and manholes when repair work is done and when waste generated from repair work is not removed completely” and “zinc may be present as many of the fillings on electrical equipment and cables are galvanized” (Pacific Gas and Electric Company 2015a, b, c, d, e). Major utilities also referenced polychlorinated biphenyls (PCBs) within their pollution prevention plans and noted that they may be present if utility vault infrastructure is older than 1980 (the national ban on PCBs was in 1979). However, both the Southern California Edison (SCE) and PG&E plans, for example, state that utility vault water encountering these types of equipment must be hauled away (not discharged) if oil is present (Pacific Gas and Electric Company 2015a, b, c, d, e; Southern California Edison 2015).

Since urban runoff is thought to be the main source of water within utility vaults, it is likely that the utility vaults (via stormwater intrusion) could contain various contaminants including PCBs, organochlorine pesticides, polycyclic aromatic hydrocarbons (PAHs), polybrominated diphenyl ethers (PDBEs), dioxins, volatile organic compounds (VOCs), pyrethroids, and/or metals, as these are commonly found in stormwater runoff (Gilbreath and McKee 2015; Zgheib et al. 2012). PCBs in San Francisco Bay, for example, are high enough to adversely impact wildlife and human health; sources of PCBs should be understood and controlled if feasible (Davis et al. 2007). Simply measuring these priority pollutants and reporting to the State Water Resources Control Board is an effort that will likely not have comprehensive immediate review, highlighting a need for this earlier analysis.

The purpose of this research is to analyze utility vault water for the 126 priority pollutants. To our knowledge, there are no reported studies in the literature of contaminant concentrations in utility vaults, making this an important characterization of the potential of utility vault water to contribute to surface water pollution. This research is also driven by the need to answer the SWRCB question in the 2014 NPDES Utility Vault Permit:

Do utility vaults and underground structures have a reasonable potential to contribute to an exceedance of water quality objectives if all provisions of NPDES No. CAG990002 (Utility Vault and Underground Structure Surface Water Discharge Permit) are followed?

Accordingly, we analyzed data on pollutant concentrations in water discharged from utility vaults owned and operated by the largest utility company in California, Pacific Gas and Electric Company (PG&E). These samples were collected as part of PG&E’s compliance with the NPDES Utility Vault Permit to meet the provisions of Characterization Study 1. We compared pollutant concentrations in utility vault water to water quality criteria. We also compared the data with values reported in the literature for stormwater. Finally, we calculated the overall impact of pollution from utility vault water by calculating potential loads, defined as the total mass of a pollutant discharged from utility vault water over the course of a year (e.g., Meals et al. 2013). We compared the potential load from all utility vaults in regions that drain into San Francisco Bay (Bay) to loads from other sources (e.g., agricultural and industrial loads) to gauge the relative impact of utility vault water discharges.

## Methods

### Regulatory requirements

Section 402 of the Federal Water Pollution Control Act (also known as the Clean Water Act), requires NPDES permits for point-source discharges. The California SWRCB, by the delegation of the Environmental Protection Agency (EPA), implements the NPDES program by issuing a variety of discharge permits for point source discharge into waters of the United States. Operators or organizations (e.g., utility companies) discharging from Utility Vaults and Underground Structures (hereafter Dischargers) into US waters must acquire an NPDES Permit (California State Water Resources Control Board 2014a). Historically, within the NPDES Utility Vault Permit, the SWRCB has exempted Dischargers from meeting water quality effluent limitations required under the Policy for Implementation of Toxic Standards for Inland Surface Waters, Enclosed Bays, and Estuaries of California (SIP) (California Environmental Protection Agency 2005). The SIP applies to discharges of toxic pollutants into waters of the state, which can be enforced through NPDES permits under the State’s Porter-Cologne Water Quality Control Act and the Clean Water Act.

The practice of issuing exemption from the SIP has recently come into question. In the 2001 and 2006 utility vault dewatering permits, SWRCB required collection of preliminary data to assess potential presence of a limited number of constituents (California State Water Resources Control Board 2001, 2006). Then, in 2014, the SWRCB deemed the collected data from the 2006 NPDES Utility Vault Permit “insufficient” to appropriately characterize utility vault water for potential pollutants (California State Water Resources Control Board 2014a). In the revised 2014 NPDES Utility Vault Permit, the SWRCB required Dischargers to perform multiple studies, including the 2-year “Characterization Study 1” to sample utility vaults within their service territory for all 126 priority pollutants. These priority pollutants are a subset of the toxic pollutants regulated by the EPA; the list of priority pollutants focuses on chemicals frequently found in waters and that have published analytical methodologies.

### Sampling locations and collection

Data utilized for this study were collected by PG&E as a provision of its permit coverage under WQO-2014-0174 (the 2014 NPDES Utility Vault Permit) and reported in its 2015/2016 Annual Monitoring Reports, within Appendix C (Pacific Gas and Electric Company 2016a, b, c, d). The data were collected in the 2015/2016 monitoring year (November–May) and represent the first year of data collection for Characterization Study 1 as required by the 2014 NPDES Utility Vault Permit. Pacific Gas and Electric Company has a service area of over 70,000 mile^2^, stretching from Bakersfield, CA through Eureka, CA (Pacific Gas and Electric Company 2016e). This expansive service territory spans five Regional Water Quality Control Board (RWQCB) boundaries (regions 1, 2, 3, 5, and 6), allowing for widespread characterization of utility vault water from a variety of utility vault structure types and locations within California. The RWQCB boundaries are set by the SWRCB to regulate water quality across watershed basins within California.

PG&E defined the types of utility vaults within its service area in its Pollution Prevention Plans: standard utility vaults (containing electrical or gas equipment); pull/switch boxes (containing only wires); substation utility vaults (a standard utility vault or pull/switch box associated with an enclosed substation); and automatic sump-pumped utility vaults (structure equipped with an automated sump-pump, oil sensor and float switch) (Pacific Gas and Electric Company 2015a, b, c, d, e).

Criteria for selecting utility vaults to be sampled included identifying utility vaults from each structure type within each RWQCB region that contained a sufficient quantity of utility vault water for sample collection. When possible, utility vaults that had been sampled in 2014 and 2015 were chosen due to sampling logistics and to allow for trend monitoring. Once all four structures were sampled, remaining samples were taken from standard utility vaults because it is the most common underground structure type. In the event that one of the identified structure types did not contain water, or could not be found, remaining samples were taken from the remaining utility vault types. All sampling occurred within the rainy season from October 2015 through May of 2016 (Pacific Gas and Electric Company 2016a, b, c, d).

**Fig. 2:**
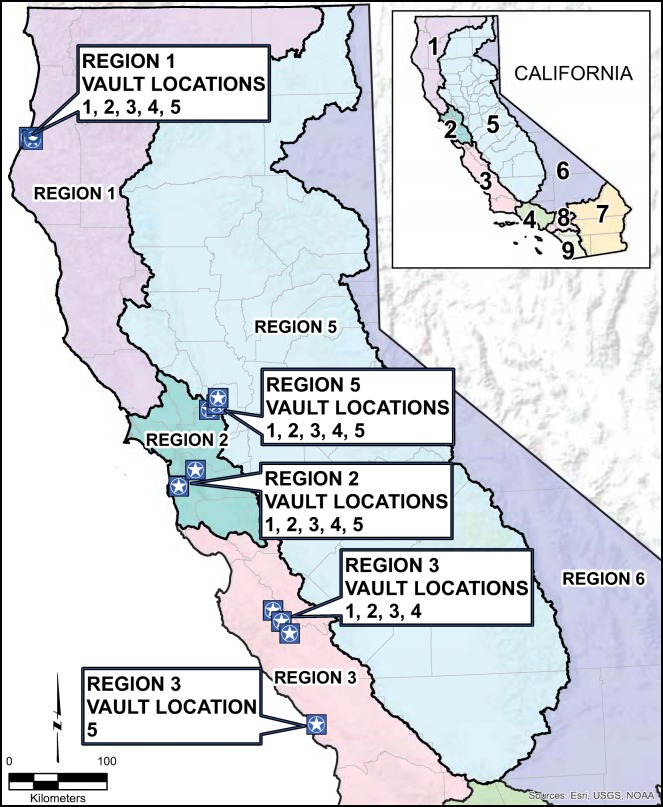
PG&E Vault sampling locations were located in the California Regional Water Quality Control Board Regions 1, 2, 3, and 5 (Pacific Gas and Electric Company 2016a, b, c, d). California is divided into 9 water quality control boards (see inset), for purposes of protecting and regulating water quality in regional water basins

PG&E collected a total of 20 utility vault water discharge samples from 20 different utility vaults spanning four Regional Water Quality Control Board Regions (1, 2, 3, and 5), as seen in Fig. 2. One field duplicate was collected in each of these Regional Water Quality Control Board Regions for a total of 4 duplicate samples, or 20% of the total number of samples. No samples were collected in the Lahontan Regional Water Quality Control Board (region 6) due to limited rainfall, sandy soils, and remote utility vault placement (Pacific Gas and Electric Company 2016a, b, c, d, e, f)*.*

The provisions of the 2014 NPDES Utility Vault Permit require that all samples be collected after Best Management Practices (BMPs) are implemented, which allows for representative discharge characterization. The BMPs implemented by PG&E call for the utility vault water to be first inspected for odors, unusual discoloration, cloudiness, product sheen, and any other evidence of chemicals, a practice highlighted as industry standard (California State Water Resources Control Board, 2014a). Only water free of these contaminants can be discharged as per PG&E’s Pollution Prevention Plans; additionally, all PG&E utility vault water appropriate for discharge is passed through a filter sock prior to release. PG&E utilizes the Pure Filter Sock, which includes a 4-layer stage filter with a 1 μm mesh size (Fig. 1) capable of pumping water through at 100 gal per minute (Pacific Gas and Electric Company 2015a, b, c, d, e; Pure Filter Solutions 2016). The use of a filter sock regardless of water clarity is slightly more stringent than typical industry practices, as many other utilities only use a filter sock if the water is cloudy, smelly, or oily (e.g., Sacramento Municipal Utility District 2015; Southern California Edison 2015). Accordingly, PG&E’s BMPs may be more protective than the industry as a whole and could lead to conservative estimates of the impact of contamination from utility vaults.

Additionally, there were two pre-BMP duplicate samples collected during the PG&E data collection study, as per the conditions listed within the sampling plan. The pre-BMP samples were collected from utility vaults containing automated sump-pumps, which would remove water from the utility vault under emergency conditions (e.g., the equipment was in danger or the vault was about to overflow). More typically, when these vaults are dewatered to access the infrastructure, standard dewatering and best management practices are used (i.e., a filter sock is employed). For purposes of the data analysis within this study, pre-filter sock samples from automated sump-pump vaults are reviewed in the results but were omitted from the tables and calculations. These pre-filtration samples are not included in the calculations because they are not representative of typical utility vault water discharge quality. Furthermore, utilizing only post-filtration samples provides consistency within the data set.

Samples were collected by lowering a submersible pump about one-foot from the bottom of the utility vault and taking a grab-sample of the discharge water from the end of the filter sock. Utility vault water was discharged through the filter sock for at least 30 s prior to sampling to fully saturate the filter sock (Pacific Gas and Electric Company 2016a, b, c, d). Samples were collected directly into sample containers supplied by the laboratory. Due to the nature of the sampling scheme, samples generally came from the middle to lower part of the utility vault, but no attempt was made to look at vertical heterogeneity. Many of the utility vaults were quite small; for 23% of the utility vaults discharged in the 2015–2016 monitoring year, the discharge volume was less that 400 L, which was about the volume of a deep bathtub. As a result, we did not expect a lot of variation within the utility vaults, but this is an area for further investigation.

As appropriate, to avoid contamination, select samples were collected by PG&E (and its contractors) using EPA Clean Hand-Dirty Hands Method 1669 (Environmental Protection Agency 1996a). Only one sample bottle at a time was opened to prevent ambient interference. No in-field (post BMP) filtration occurred. Any samples requiring filtration prior to analysis (e.g., hexavalent chromium analysis) were filtered by [and at] the analytical laboratory (Pacific Gas and Electric Company 2016a, b, c, d).

All data reported within this study are from the “primary” samples, marked as “A” within the data set. Duplicate samples were collected for quality control (e.g., calculation of the relatively percent difference) but not used in the calculations. One exception was sample V5-R5-A, which was noted as having a broken bottle during shipment. Accordingly, results from the duplicate (V5-R5-B) were utilized for data analysis.

### Analytical methods and reporting

All samples were analyzed by Eurofins/Calscience labs in Southern California, using the EPA methods specified within the 2014 NPDES Utility Vault Permit and listed within PG&E’s Characterization Study Plan (California State Water Resources Control Board 2014a; Pacific Gas and Electric Company 2015f). In general, instrumentation used for the analysis is as follows: metals were analyzed using inductively coupled mass spectroscopy; pesticides and polychlorinated biphenyls were analyzed using gas chromatography; polycyclic aromatic hydrocarbons, volatile organic compounds, and semi-volatile organic compounds were analyzed using gas chromatography mass spectroscopy and mercury samples were analyzed using cold vapor atomic fluorescence spectrometry. Table 1 shows the analytical methods and the general range of detection limits achieved; sample-specific detection limits shown in the Online Resource.

**Table 1:** Methods and range of detection limits for constituents analyzed in utility vault water. Specific detection limits for each sample are listed in the Online Resource

Constituent	Units	Analytical test method	Method detection limit range
pH	SU	hand-held pH Pen	n/a
Metals (Sb, As, Be, Cd, Cr, Cu, Pb, Ni, Se, Ag, Tl, Zn)	μg/L	USEPA Method 6020	0.0898–0.479
Mercury	ng/L	USEPA Method 1631E	0.113
Hexavalent chromium	μg/L	USEPA Method 7199	0.067–1.3
Oil and grease (O&G)	mg/L	USEPA Method 1664A HEM	0.80
Organochlorine pesticides	μg/L	USEPA Method 8081A/USEPA Method 608	0.00050–0.30
Polycyclic aromatic hydrocarbons (PAHs)	μg/L	USEPA Method 8270C/USEPA Method 8270C SIM PAHs	0.0046–2.3
Polychlorinated biphenyls (PCBs)	μg/L	USEPA Method 8082	0.060–0.15
Total cyanide	μg/L	Standard Method 4500-CN E	7
Total hardness (as CaCO3)	μg/L	Standard Method 2340C	990
Total suspended solids (TSS)	μg/L	Standard Method 2540D	830
TPH-diesel (TPH-d)	μg/L	USEPA Method 8015B (M)	8.0
TPH-gas (TPH-g)	μg/L	USEPA Method 8015B (M)	48
Volatile organic compounds (VOCs)	μg/L	USEPA Method 8260/USEPA Method 8270C B	0.14–8.0
Semi-volatile organic compounds (SVOCs)	μg/L	USEPA Method 8270C	0.0018–7.0
2,3,7,8-Tetrachlorodibenzo-p-dioxin	pg/L	USEPA Method 8290	0.286–0.907

*mg/L* milligrams/liter, *μ*g/L micrograms/liter, *ng/L* nanogram/liter, *pg/L* picogram/liter, *SU* standard units, *USEPA* U.S. Environmental Protection Agency

Detection limits in this study were set to comply with the water quality criteria set by the Utility Vault Discharge Permit (Table 1, Online Resource). The water quality criteria reflect the most stringent criteria needed to protect aquatic life from acute effects or to protect human health from significant risk (California State Water Resources Control Board 2014b, Appendix G8). Many detection limits were consistent with previous stormwater studies and were low enough to allow detection of the pollutants at the level that would be expected in stormwater. For example, the detection limit for total mercury (HgT) in this study was 0.113 ng/L (Online Resource). That concentration was comparable to the detection limit of 0.16 ng/L reported by McKee et al. (2017) for HgT in the Guadalupe River Watershed, which drains into San Francisco Bay.

However, some pollutant detection limits in the categories of PAHs, PCBs, and chlorinated pesticides were above the water quality criteria. The SWRCB authorized the use of the minimum detection levels listed within the SIP when the water quality criteria listed within the 2014 NPDES Utility Vault Permit were not met. Only 11 of the 126 priority pollutants sampled Nov, 2015 to Jan, 2016 met neither of these criteria; these pollutants were accordingly resampled. For example, dibenz(a,h)anthracene initially had a detection limit of 1.2 μg/L, which was above the water quality criteria of 0.049 μg/L. After re-sampling, the detection limit was 0.0047 μg/L, which met the water quality criteria. In contrast, dieldrin initially had a detection limit of 0.014 μg/L, which was above the water quality criteria of 0.00014 μg/L. After re-sampling, the detection limit was 0.00050 μg/L, which met the SIP, but not the water quality criteria. After re-sampling, all constituents met either the limits set in the 2014 NPDES Utility Vault Permit or the SIP. The one exception was 2,3,7,8-TCDD [dioxin], which did not have minimum detection limit in the SIP; the standard laboratory detection limit of < 1 picogram per liter was used as confirmed with the SWRCB.

Overall quality assurance/quality control for the PG&E preliminary data set was good. Relative percent difference, calculated as the difference between the primary sample and the duplicate sample divided by the average of the two samples (Ohio EPA 2012), averaged 9% for region 1 samples, 8% for region 2 samples, 15% for region 3 samples, and 13% for region 5 samples. The greatest range in the relative percent difference was seen in region 3, with the relative percent difference ranging from 0 to 53%, with the highest variation seen in the Zn results. These ranges are typical of variability between field samples.

The 2014 NPDES Permit requires the reporting of j-flagged data, where the exact concentration of the analyte is an estimate, but we can say with 99% confidence that the analyte is present in the sample. Specifically, data receive a j-flag if they are higher than the method detection limit (calculated as the standard deviation of at least 7 replicate low-level spikes times the one-tailed critical t value from the Student’s *t* distribution at 99% confidence) but lower than the analytical reporting limit (typically 2–5 times the method detection limit) (Calscience Environmental Laboratories Inc. 2014). Essentially, method detection limits are the lowest concentration of a constituent that can be measured by the defined method and instrumentation, whereas reporting limits are thresholds set by the analytical labs where the numeric values are believed to be [reasonably] accurate (California Department of Public Health 2005; Wisconsin Department of Natural Resources 1996). The j-flagged data thus indicate concentrations that are measurable but potentially not accurate.

### Loading calculations

To determine if the utility vault water contaminants show a “reasonable potential” for addition, we calculated the annual load of Cu and Zn from utility vaults. We calculated loads by multiplying the median concentration of Cu and Zn in the utility vault samples by estimates of the average annual discharge volume. We used the median concentration, rather than the mean, because the median is resistant to extreme values. Furthermore, for utility vault water, the extreme values are probably not representative of all utility vaults because each utility vault contains a different blend of water accumulated from multiple different inputs, including storms of various sizes. For example, one utility vault may be emptied after it has accumulated water from one or many small storms whereas another may be emptied after it accumulates water from a single large storm. This situation differs from typical stormwater analyses, where high flows tend to mobilize more pollutants than lower flows, making the extreme storms most relevant for calculating loads (Gilbreath and McKee 2015; Howell et al. 2011). The focus was on Cu and Zn because they were the only two constituents that exceeded the defined regulatory limits—either the water quality objectives set within the 2014 NPDES Utility Vault Permit or the minimum levels listed within the SIP.

The goal of the first set of analyses was to compare utility vault water loads with other types of loads into San Francisco Bay. The Bay drains 40% of the State of California (Conomos et al. 1985), meaning many water pollutants end up there eventually. Furthermore, these is an existing body of literature (e.g., Davis et al. 2000) on loads to this estuary so it was possible to place utility vault water loads in context. Accordingly, the first set of analyses used discharge data from RWQCB Regions 2 and 5 because these watersheds drain into the Bay. The second set of analyses used estimated utility vault discharges from across California to evaluate the broader contribution of utility vault discharges to the annual Cu and Zn load into all State surface waters.

To estimate the total utility vault discharges across the state, we used historical and current enrollee data from NPDES No. CAG990002, the NPDES Utility Vault Permit. The Utility Vault General NPDES Permit was originally issued by the SWRCB on August 15, 1996 and was revised in 2001, 2006, and again in 2014 (California State Water Resources Control Board 2001, 2006, 2014a). Utilizing the “General Order Report” tool provided by the SWRCB, we tallied the number of Dischargers under each iteration of the permit. The 2014 Utility Vault Permit requires each Discharger to provide an estimate of the annual volume discharged, which is then reported in each of their annual monitoring reports.

At PG&E, the discharge volume is estimated at the time of discharge using categories: < 100 gal, 100–1000 gal, 1000–5000 gal, 5000–10,000, or greater than 10,000 gal, but the exact volume is not recorded. Using the PG&E data, we took the midpoint of each category, converted it to liters, multiplied by the total number of PG&E discharge events in that category and then summed all of the categories to obtain the approximate total volume discharged (Laurin 2016). Loads for PG&E were calculated as the total volume discharged times the median or maximum metal concentration. To expand and gain a statewide estimate, we multiplied the estimated PG&E loads by the total number of Dischargers as shown by the SWRCB’s “General Order Report” tool under the NPDES Utility Vault Permit.

## Results

### Detection of priority pollutants

Twenty-one priority pollutants were detected in the PG&E utility vault water discharges using the analytical methods specified in the 2014 NPDES Utility Vault Permit or the SIP (Table 2). Of the 21 detected constituents, all except Zn and Ni had j-flagged data (Tables 2 and 3). If the low-level j-flagged results are omitted, only 14 results show measurable concentrations of the analytes. Of these 21 detections, two constituents (Cu and Zn) exceeded the water quality criteria set within the 2014 NPDES Utility Vault Permit and/or the SIP. The thresholds for the SIP were used as a comparison for the utility vault water data only when the methods of analysis were unable to meet the limits required in the 2014 NPDES Utility Vault Permit.

**Table 2:** Twenty-one priority pollutants were detected in utility vault water collected by PG&E for compliance under the 2014 NPDES Vault Permit Study 1 (Pacific Gas and Electric Company 2016a, b, c, d). J-flagged data (J) indicate that concentrations are above the method detection limit, but lower than the reporting limit. Water quality criteria (WQC) for metals varies based on total hardness (as CaCO3), as identified in the 2014 NPDES Vault Permit and shown in Table 3 (California State Water Resources Control Board 2014, Table G-4). When WQC were not available, or were too low for analysis, criteria from the State Implementation Policy (SIP) were used (California Environmental Protection Agency 2005, Appendix 4). The symbol # indicates that permit benchmark criteria were used because no water quality criteria were set within the 2014 NPDES Vault Permit. HD indicates that detections were inconsistent with the chromatograph pattern of the lab’s reference standard

	Frequency of detections (including j-flagged results)	Frequency of results above analytical reporting limit (i.e., non-j-flagged)	Water quality criteria (NPDES Vault Permit)	State implementation policy (SIP) criteria	Frequency of results above water quality criteria (all were non-j-flag)	Maximum concentrations
General minerals [mg/L]						
Solids, total suspended	10%	10%	400#	n/a	0%	10
Metals [μg/L]						
Antimony	95%	50%	4.3	0.5	0%	99.9
Arsenic	70%	60%	0.3	1.0	0%	89
Cadmium	25%	0%	hardness based	0.25	0%	0.425 J
Copper	100%	95%	hardness based	0.5	25%	791
Lead	90%	25%	hardness based	0.5	0%	7.85
Mercury	90%	85%	51	0.2	0%	0.0104
Nickel	100%	100%	hardness based	1	0%	7.57
Selenium	70%	15%	hardness based	1	0%	7.79
Zinc	100%	100%	hardness based	1	15%	386
Chromium	100%	50%	hardness based	0.5	0%	13.1
Chromium, hexavalent	60%	25%	16	5	0%	10
Oil and grease [mg/L]						
Oil and grease	15%	10%	25#	n/a	0%	1.5
Total petroleum hydrocarbons (TPH) [mg/L]						
TPH as diesel	50%	5%	2#	n/a	0%	54 HD
Volatile organic compounds (VOC) [μg/L]						
1,1,1-Trichloroethane	5%	0%	n/a	0.5	0%	0.36 J
Acetone	5%	0%	n/a	n/a	0%	5.8 J
Chloroform	5%	0%	n/a	0.5	0%	0.43 J
Chloromethane	5%	0%	n/a	0.5	0%	0.40 J
Dibromochloromethane	5%	0%	34	0.5	0%	0.41 J
Tert-butyl alcohol (TBA)	5%	0%	n/a	n/a	0%	4.8 J
Tetrahydrofuran	5%	5%	n/a	n/a	0%	6.1

**Table 3:** Comparison of metal concentrations in utility vault water data to the corresponding hardness-based water quality criteria (WQC). Note that only metals that have hardness-based criteria are shown, and J-flagged data (J) indicate that concentrations are lower than the reporting limit. Non-detected concentrations are reported as < method detection limit. Italicized cells indicate results above the water quality criteria listed within the 2014 NPDES Vault Permit. Table populated by data collected by PG&E for compliance under the 2014 NPDES Vault Permit (Pacific Gas and Electric Company 2016a, b, c, d)

Sample ID	Total hardness (as CaCO3) (mg/L)	Chromium (μg/L)		Copper (μg/L)		Lead (μg/L)		Nickel (μg/L)		Selenium (μg/L)		Zinc (μg/L)	
		WQC	Analytical result	WQC	Analytical result	WQC	Analytical result	WQC	Analytical result	WQC	Analytical result	WQC	Analytical result
V1-R1-EFF-A	130	2400	0.925 J	21	*791*	140	2.43	660	1.60	N/A	< 0.168	170	*227*
V2-R1-EFF-A	350	3100	0.545 J	27	15.8	200	0.409 J	840	2.50	N/A	< 0.168	220	96.4
V3-R1-EFF-A	87	1400	0.793 J	11	5.1	57	0.332 J	370	1.56	N/A	< 0.168	94	*386*
V4-R1-EFF-A	220	3100	0.483 J	27	9.56	200	0.264 J	840	4.95	N/A	0.264 J	220	168
V5-R1-EFF-A	47	900	1.27 J	6.6	4.44	30	0.462 J	240	1.13	N/A	< 0.168	61	18.3
V1-R2-EFF	350	3100	0.490 J	27	3.25	200	2.78	840	4.56	N/A	< 0.168	220	74.0
V2-R2-EFF	330	3100	0.424 J	27	3.44	200	7.85	840	7.57	N/A	0.228 J	220	63.1
V3-R2-EFF	170	2400	0.467 J	21	9.66	140	0.825 J	660	3.94	N/A	0.312 J	170	99.5
V4-R2-EFF	190	2400	0.551 J	21	20.5	140	0.219 J	660	1.59	N/A	0.504 J	170	34.2
V5-R2-EFF	220	3100	3.13	27	10.4	200	1.27	840	1.81	N/A	0.266	220	20.3
V1-R3-EFF	270	3100	1.35	27	*88.0*	200	0.125 J	840	3.21	N/A	0.524 J	220	*307*
V2-R3-EFF	400	3100	0.915 J	27	1.76	200	0.189 J	840	5.31	N/A	0.185 J	220	106
V3-R3-EFF	320	3100	1.46	27	13.5	200	< 0.0898	840	1.92	N/A	0.297 J	220	20.1
V4-R3-EFF	260	3100	0.797 J	27	8.19	200	< 0.0898	840	4.59	N/A	< 0.168	220	16.8
V5-R3-EFF	330	3100	2.07	27	*116*	200	1.73	840	1.74	N/A	6.07	220	26.2
V1-R5-A	350	3100	1.54	27	6.36	200	0.0927 J	840	1.70	N/A	0.547 J	220	99.1
V2-R5-A	190	2400	2.73	21	*51.0*	140	0.175 J	660	2.75	N/A	0.694 J	170	166
V3-R5-EFF-A	280	3100	2.63	27	0.284 J	200	0.259 J	840	1.44	N/A	0.468 J	220	89.2
V4-R5-A	140	2400	2.62	21	12.4	140	0.680 J	660	1.01	N/A	0.841 J	170	15.7
V5-R5-B	48	900	13.1	6.6	*7.91*	30	0.398 J	240	1.09	N/A	7.79	61	20.8

The remaining 105 priority pollutants were not detected (Online Resource). Data from the selected resampling effort also come back with non-detect results, consistent with the results from the first sampling effort, but with a lower reporting limit to meet the conditions of the SIP per conversations with the SWRCB.

Metals were the most commonly detected constituents (Table 2), with antimony, arsenic, copper, lead, mercury, nickel, selenium, zinc, chromium, and hexavalent chromium found in at least one of the samples at levels above the analytical reporting limit. Various volatile organic compounds (VOCs) were also detected in the samples, but only tetrahydrofuran was found at levels above the analytical reporting limit. For the 21 detected constituents, all except two (Zn and Ni) had at least one detected analytical result reported with a j-flag, meaning that the constituents were present, but levels of the constituents were often too low for quantification (Table 2).

The maximum concentrations of Cu and Zn (Table 2) were 791 μg/L and 386 μg/L, values that exceeded the hardness-based water quality criteria (Cu of 21 μg/L and for Zn was 94 μg/L), where the water quality criteria corresponding to the appropriate hardness are given. Copper and Zn water quality criteria (California State Water Resources Control Board 2014a) range from 6.6 to 27 μg/L for Cu and 17 to 220 μg/L for Zn, based on the CaCO_3_ concentration in the effluent. The mean Cu concentration was 62.0 μg/L, substantially higher than the median Cu concentration of 9.66 μg/L, reflecting influence of the single sample with 791 μg/L of Cu. That Cu sample was a factor of almost 7 higher than the next highest result. However, the other high Cu values still exceed the water quality criteria by factors of up to 4. The minimum Cu concentration was 1.76 μg/L. The mean Zn concentration was 103 μg/L, and the median was 81.6 μg/L, reflecting the presence of multiple relatively high Zn results in the data set. The Zn results also exceeded the water quality criteria by up to a factor of 4. The minimum Zn concentration was 15.7 μg/L.

### Effects of filtration, location, and type of structure

Two samples were collected prior to the use of a filter sock from utility vaults containing automated sump-pumps. In region 2, total suspended solids (TSS) in the pre-filter sample were higher (6 mg/L) than in the post-filtration sample (< 0.83 mg/L). A corresponding decrease in pollutant concentrations (for those with quantifiable concentrations) was generally observed. For example, Cu declined from 6.74 to 3.25 μg/L in the post-filtration sample. Similarly, lead declined from 7.92 to 2.78 μg/L and Zn from 89.3 to 74.0 μg/L. In region 5, both the pre- and post-filter sample had TSS concentrations < 0.83 mg/L and the prevalence of j-flagged data made it hard to distinguish any trends.

Exceedances of the water quality criteria in the 2014 NPDES Utility Vault Permit for Cu and Zn were seen within all but the San Francisco Bay Area (region 2) (Table 3). Region 2 contained no water quality exceedances and had the lowest overall range of concentrations of Cu and Zn across the regions (3.25–20.5 μg/L and 20.3–99.5 μg/L, respectively). Utility vaults within the North Coast (region 1) reported the highest results of both Cu and Zn (4.44–791 μg/L and 18.3–386 μg/L, respectively), although median concentrations of Cu were comparable to the other regions. The median concentration of Cu across all regions (1, 2, 3, and 5) was very similar (9.56 μg/L, 9.66 μg/L, 13.5 μg/L, and 10.2 μg/L, respectively). Median concentrations of Zn across all regions varied further, ranging from 26.2 μg/L for Region 3 to 168 μg/L for region 1. One organic was detected in each region, except region 5, which had four organic detections.

There was no apparent influence of the type of structure (e.g., standard utility vaults, pull/switch boxes, substation utility vaults, or automatic sump-pumped utility vaults) on pollutant concentrations within or across regions. Within each structure type, there was a fair bit of variation in contaminant concentrations. For example, in region 3, three standard utility vaults were sampled with Zn results ranging from 16.8 to 106 μg/L.

### Quantifying utility vault water discharge volume

While we know that there were 2105 utility vault discharges performed by PG&E in the 2015/2016 monitoring year, it is unknown how many utility vaults there are within California and also how many of them are routinely discharged. As a result, PG&E data were used to calculate loads and as a reference point for approximation statewide (Pacific Gas and Electric Company a, b, c, d, f). Within 2015, utility vault water discharges at PG&E ranged from approximately 200 to 40,000 L, with most discharges being between 400 and 4000 L (Pacific Gas and Electric Company 2016a, b, c, d, f). It also appears that 98% of PG&E’s annual utility vault discharges are less than 20,000 L, meaning that discharges are typically less than the size of a small swimming pool. Total estimated annual discharge from PG&E’s utility vaults was 7000 m^3^.

When the SWRCB “General Order Report” tool was used to obtain the number of enrollees under each iteration of NPDES No. CAG990002, 56 enrollees were listed as retaining coverage under the 2014 NPDES Utility Vault Permit and 74 organizations held coverage under the previous NPDES Utility Vault Permit iteration (California State Water Resources Control Board 2016). Discharger calculations were based on the number of enrollees under the 2006 NPDES Utility Vault Permit because it contained the largest number of permit holders across all of the various NPDES Utility Vault Permits. The reduction in current permit enrollees could be due to historical Dischargers not yet filing for coverage under the new permit, as there are quite a few “active enrollees” still under the 2006 NPDES Utility Vault Permit.

Enrollees in NPDES No. CAG990002 are operators and organizations (e.g., utility companies) of varying sizes and spans. Examples of large (state-spanning) utility companies include PG&E, AT&T, Verizon, Pacific Bell Telephone Company, and Southern California Edison. Small (local utility companies) include Santa Clara Valley Transportation Authority, Glendale Water & Power, Veolia Energy, Sonic Telecom, and Redding Electric Utility (California State Water Resources Control Board 2018). PG&E is the Nation’s largest electric and gas utility company and one of the largest utility companies in California. Based on size and distribution, it is expected that by multiplying PG&E’s results by 74 (to estimate state-wide loadings) would likely overestimate pollutant loading because most utility companies listed are smaller than PG&E.

### Loads

PG&E’s utility vault water discharge data from RWQCB Regions 2 and 5 (Fig. 2) were selected to focus on discharges with the potential to contaminate the Bay. When the total volume of PG&E 2015/2016 discharges into the Bay was multiplied by the median Cu and Zn concentrations from the entire data set (to provide the most accurate concentration estimates), median annual loads of Cu and Zn were approximately 0.06 kg/year and 0.5 kg/year, respectively. When the total discharge volume to these two regions was multiplied by the maximum concentrations of Cu and Zn (791 μg/L and 386 μg/L, respectively) from this study Cu and Zn loads were approximately 5 kg/year and 2 kg/year, respectively.

Next, PG&E's utility vault water discharge data from RWQCB Regions 1, 2, 3, and 5 were selected to evaluate PG&E's discharge contribution statewide. The total volume discharged statewide (~7x10^6^L) was only slightly higher than the total volume (~6x10^6^L) discharged in Regions 2 and 5 alone. When the total discharge volume to all regions was multiplied by the median Cu and Zn concentrations, median loads into California surface waters for all of PG&E’s utility vault water discharges were 0.07 kg/year for Cu and 0.6 kg/year for Zn (Table 4). Using the total discharge volume from all regions and highest concentrations of Cu and Zn (791 μg/L and 386 μg/L, respectively), Cu and Zn maximum loads into California surface waters were approximately 5 kg/year and 3 kg/year, respectively.

**Table 4:** Estimated loads of total copper and zinc from utility vaults and underground structures. Category shows the amount of vault water discharged and is aggregated from PG&E records, where field crews use categories to estimate volumes of vault water released. For this calculation, the midpoint (in L) was multiplied by the median concentration from all samples and the reported number of vault discharges to calculate the total annual loading for all PG&E vault discharges. To obtain statewide load estimations for all utility vault discharges, the total PG&E vault loading data was multiplied by 74, which was the number of 2006 NPDES Utility Vault permit holders (California State Water Resources Control Board 2016). Data pulled from PG&E’s characterization study data (Pacific Gas and Electric Company 2016a, b, c, d)

Constituent	Category (gallons)	Mid point (liters)	Median concentration (μg/L)	Average loading of one PG&E vault (g)	Reported number of total PG&E vault discharges (2015–2016 monitoring season)	Total annual median loading for PG&E vault discharges (kg/year)	Estimated total annual median loading for all utility vault discharges (kg/year)
Copper	< 100	189	9.66	0.002	484	0.001	0.1
	100–1000	1703	9.66	0.016	1113	0.02	1
	1000–5000	7571	9.66	0.073	462	0.03	3
	5000–10,000	28,391	9.66	0.274	46	0.01	1
	Sum	37,854	39	0.366	2105	0.07	5
Zinc	< 100	189	81.6	0.02	484	0.01	1
	100–1000	1703	81.6	0.14	1113	0.2	10
	1000–5000	7571	81.6	0.62	462	0.3	20
	5000–10,000	28,391	81.6	2.32	46	0.1	8
	Sum	37,854	326	3.09	2105	0.6	40

When the median loads from PG&E’s utility vault data were multiplied out across all 74 2006 NPDES Utility Vault Permit holders, estimated total median loads for all utility vaults across California (i.e., utilizing utility vault water data from RWQCB Regions 1, 2, 3, and 5) were 5 kg/year for Cu and 40 kg/year for Zn (Table 4). The same calculations were made using the maximum concentrations of these two metals, but this worst-case scenario is not representative of our data set. Statewide maximum loads for all utility vault discharges are 400 kg/year for Cu and 200 kg/year for Zn.

## Discussion

### Composition and concentrations indicate stormwater source

Because water that accumulates in utility vaults comes primarily from stormwater runoff and/or groundwater intrusion (California State Water Resources Control Board 2014a), we had hypothesized that the composition of utility vault water would mirror that of stormwater. A wide variety of pollutants may be present in stormwater. For example, Zgheib et al. (2008) identified 88 different constituents from 13 different chemical families that are likely to present in stormwater runoff from urban areas. In a subsequent study, Zgheib et al. (2012) detected 55 different substances in Paris stomwater, but only 8 of those were likely to cause adverse effects: Pb, Cu, Zn, phenanthrene, pyrene, benzo[*a*]anthracene, chrysene, and dibenzo[*a,h*] anthracene. In the present study, only 21 of the 126 priority pollutants analyzed were detected (Table 2). If leaking equipment within the utility vaults was a source, we would have expected much higher concentrations than were measured. Only Cu and Zn exceeded the water quality criteria (in some samples), which were metals already identified by Zgheib et al. (2012) and dischargers as being potentially problematic.

Median concentrations of Cu and Zn in utility vault water were generally lower than, or in the range of, concentrations reported in stormwater. For example, median Cu and Zn concentrations in utility water in this study were 9.66 and 81.6 μg/L, substantially lower than the median concentrations in Paris stormwater of 55 and 270 μg/L, respectively (Zgheib et al. 2012). Median Cu and Zn concentrations in utility vault water were comparable to the range of stormwater concentrations measured in a local study of urban runoff from Hayward, CA (McKee and Gilbreath 2015). That study, which focused on the smaller urban San Francisco Bay Area outfalls over 4 years (from water years 2007–2010) reported that Cu concentrations ranged from 2.3 to 50 μg/L and Zn concentrations ranged from 2.4 to 280 μg/L (McKee and Gilbreath 2015), which is similar to low and averaged utility vault water data. In another San Francisco Bay tributary, which drains a historical mining area, Cu concentrations ranged from 2.7 to 91 μg/L and Zn concentrations ranged from 4.6 to 350 μg/L from 2003 to 2014 (McKee et al. 2017). Although the range of concentrations points to stormwater as the source of contaminants in utility vault water, further comparison of utility vault inflow and discharge water would help validate this hypothesis.

Despite the relatively low median concentrations overall, the maximum Cu concentration in the present study (791 μg/L) stands out because it is much higher than the maximum concentration (220 μg/L) in the Paris study (Zgheib et al. 2012). It is also much higher than the maximum concentration (50 μg/L) reported during high stormwater flow in an urban tributary to San Francisco Bay in Hayward, CA (McKee and Gilbreath 2015). High Cu concentrations have previously been reported in roadway and other specific source runoff (Beck and Birch 2012; Charters et al. 2016; Huber et al. 2016). This high concentration was sampled in utility vault from RWQCB Region 1, in Eureka, along the northern California Coast, in an area near boatyards and marinas. Copper has a long history of use in the maritime industry as part of antifouling paints (Luoma and Rainbow 2008), suggesting the potential for local point sources to contribute to utility vault water contamination. However, further investigation to compare the water in this utility vault to stormwater runoff would help to rule out utility vault equipment as the source of the Cu. Such a comparison could be useful in determining whether utility vaults can be a source of Cu.

Lead was another pollutant identified by many dischargers as potentially problematic. It was detected in 90% of our samples. The concentration of lead in stormwater (along with aluminum and iron) is strongly correlated to turbidity (McKee and Gilbreath 2015). Lead from the use of gasoline in the 1980s is washed out of the San Francisco Bay watershed when storms mobilize lead-contaminated sediments (Steding et al. 2000) and is a potential source of the lead in the utility vault water. Lead released from building sidings, especially painted wood, is another potential source (Davis et al. 2001). Although worrisome because lead is neurotoxic to humans at any concentration, concentrations in the present study (range < 0.0898–7.85 μg/L, Table 3) were about an order of magnitude lower than those in Paris (< 10–129 μg/L, Zgheib et al. 2012). These concentrations were similar to the range (0.18–4.6 μg/L) reported for low flow in the Hayward tributary to San Francisco Bay and less than the range (4.5–40 μg/L) for that tributary under high flow conditions (McKee and Gilbreath 2015). Concentrations in our study were also well below the water quality criteria (Table 3).

Many of the organic pollutants in this study had non-detects, including the PAHs that were identified by Zgheib et al. (2012) as problematic in Paris stormwater. This category of pollutant is to be expected in urban run-off because PAHs are byproducts of incomplete combustion. They have also have been reported in runoff from an urban watershed that drains into San Francisco Bay, with concentrations for the sum of PAHs varying from 0.0139 to 1.400 μg/L under low flow conditions and from 1.400 to 22.6 μg/L under high flow conditions (Gilbreath and McKee 2015). With the detection limits of around 1 μg/L in the present study, we would not be able to detect the lower end of the range, but we should have been able to detect peaks indicating sources. Interestingly, when dibenzo[*a,h*] anthracene was resampled with a lower detection limit of 0.0047 μg/L, which should have allowed us to detect it at the concentrations of 0.012–0.109 μg/L reported by Zgheib et al. (2012), it was still not detected.

Most notably, PCBs were not detected in this study, despite their long history of production (1930s to the 1970s) and frequent presence in urban runoff in the San Francisco Bay Area (Davis et al. 2007). For PCBs, the method detection limits in this study were sufficient to establish that concentrations were below the levels found in some industrial and urban areas, but not low enough to fully assess the contribution from stormwater. The lack of detections of these neurotoxic organics can be partially attributed to the analytical lab’s use of the higher detection limits in the SIP. Detection limits in this study ranged from 0.060 to 0.150 μg/L (Table 1, Online Resource). This detection limit could capture PCBs if they were found at concentrations of greater than 0.1 μg/L, which would be expected for watersheds with a known industrial source (Gilbreath and McKee 2015) or in some urban areas, such as the center of Paris, which had a median concentration of 0.468 μg/L (Zgheib et al. 2011). However, in an urban tributary to San Francisco Bay in Hayward, where there was a history of industrial activity, but no known PCB source, flow weighted concentrations were much lower, averaging 0.0145 μg/L, with the lowest values during low flows (Gilbreath and McKee 2015). Similarly, total PCBs in Houston, Texas, ranged from 0.00082 to 0.094 μg/L with a mean of 0.005 μg/L (Howell et al. 2011). Concentrations of PCBs were particularly low (flow weighted average of 0.000340 μg/L) in the San Francisco Bay Delta, where the two major rivers feed the San Francisco Bay; the rivers drain mostly agricultural land and open space (David et al. 2015).

Because PCBs are a pollutant of concern, the SWRCB may want to change the method suggested in the permit, which currently recommends EPA method 8082, but EPA method 1668 would be needed to detect very low levels. That being said, there is no evidence from this study that utility vault equipment is a source of PCBs, above and beyond what is found in stormwater. Furthermore, PG&E (and other large utilities such as SCE) have standard dewatering practices, which prohibit the release of utility vault water with an oil sheen if it contains equipment with a PCB potential. In these instances, the utility vault water gets trucked to a disposal facility (Pacific Gas and Electric Company 2015a, b, c, d, e; Southern California Edison 2015).

In this preliminary study, utility vault water pollutant concentrations were generally in the range of concentrations found in stormwater. However, the use of some relatively high detection limits, as specified by the SWRCB, limited our ability to assess some organic compounds at the low end of their environmental concentrations. Further quantification of concentrations would be advisable before calculating loads for these organics. The expense of the low-level analyses for potentially problematic compounds, such as PCBs, could potentially be offset by analyzing fewer priority pollutants.

Although metals were frequently detected and found in the range expected for stormwater (Table 2), there were some notable differences between utility vault water and stormwater. For example, our maximum Cu concentration of 791 μg/L was an order of magnitude higher than the maximum reported for stormwater in an urban tributary to San Francisco Bay (McKee and Gillbreath 2015). In contrast, our maximum Pb concentration of 7.85 μg/L was an order of magnitude lower than the maximum Pb concentration reported in that same urban tributary (McKee and Gilbreath 2015). The difference in Pb concentrations may point to a distinction between stormwater and utility vault water. In the urban tributary, high stormwater flows can mobilize Pb, but utility vault water can be a composite of many different storms, including multiple low flow events that generally have lower Pb concentrations than high flow events (McKee and Gillbreath 2015). The use of the filter-sock for utility vault water discharges may also reduce the amount of Pb, which binds strongly to particles. These results suggest that Cu and Zn are the pollutants in utility vault water with the most potential for adverse effects. These two metals were detected in 100% of the samples, and it is therefore relevant to further explore their potential impacts and calculate loads.

### Sources of pollutants within stormwater

Stormwater runoff can contain metal contaminants from a variety of sources: metal [Cu and galvanized] roofs (Charters et al. 2016; Gasperi et al. 2012), scrap metal yards (Aryal et al. 2010), combined sewer system pipe erosion (Gasperi et al. 2012), suburban stormwater catchments (e.g., gutters) (Gasperi et al. 2012), and/or vehicle parts (e.g., brakes, tires and engines) (Aryal et al. 2010). Many researchers have shown that the primary source of heavy metals (specifically Cu and Zn) released during rain events is from urban roadways, due to deposition and accumulation from vehicle traffic including brake pad wear, engine parts, and tire wear (Aryal et al. 2010; Birch et al. 2015; Charters et al. 2016; Huber et al. 2016). Additionally, McKee and Gilbreath (2015) showed a correlation between turbidity and trace elements, which is reiterative of the findings surrounding road-deposited sediment as a source of heavy metals within stormwater runoff (Aryal et al. 2010; Charters et al. 2016; Gasperi et al. 2012; Zgheib et al. 2012; Zhang et al. 2015). Roadways are built and used across all land-use types, often with utility vault structures located within the line of flow, or next to the storm drain (Fig. 1).

Both Cu and Zn are strongly bound to particles (McKee and Gilbreath 2015) so we expected that the PG&E practice of using a filter sock to remove some suspended sediments prior to discharge would reduce pollutant concentrations. The reduction in concentrations of TSS, Cu, Pb, Zn, and naphthalene in the post-filter sample from region 2 (Online Resource) generally supports this hypothesis. In region 5, both pre-filter and post-filter TSS results were < 0.83 mg/L so the effects of the filter were ambiguous. It is likely that the use of the filter sock improved the quality of the discharged water when particulate loads were high, but additional pre- and post-sampling would be necessary to more fully evaluate this practice. Furthermore, PG&E utilizes stricter BMPs than some other Dischargers (e.g., filtering all utility vault water through a multi-stage filter sock, instead of conditionally filtering utility vault water), meaning that the contaminant-load may be reduced when compared to other Dischargers’ practices. Setting a uniform standard structural BMP requirement for all Dischargers within the next NPDES Utility Vault Permit could help provide consistency for reduction of sediment-bound contaminants within utility vault water statewide.

All samples collected in this study were grab samples. Although utility vaults are relatively small in size, there could be variation within the water column. Discharged volumes described from PG&E within this study were approximately 200 to 40,000 L, with most discharges being between 400 and 4000 L (Pacific Gas and Electric Company 2016a, b, c, d, f). For this study, utility vaults were sampled after 30 s of flow, from approximately a foot from the bottom of the utility vault. Sample utility vault water at the beginning, middle, and end of the discharge should be done to explore the extent of any variation; alternatively, utility vaults could be entirely be pumped into a storage container, homogenizing the contents prior to sampling.

### Toxicity of pollutants within stormwater

To evaluate toxicity, we compared Cu and Zn concentrations in utility vault water to toxicity levels for rainbow trout (*Oncorhynchus mykiss*), a native California species. This species is found across the entire state of California and is well adapted to short-term stressful living conditions, including short-term droughts (Alagona et al. 2012). It is important to note that these “species mean” Cu and Zn values were normalized by the EPA according to hardness and other water quality parameters (e.g., temperature and dissolved organic carbon) for Cu and hardness for Zn (Environmental Protection Agency 2007). Also, Cu and Zn both are naturally occurring and essential to life below toxic thresholds (Luoma and Rainbow 2008).

Copper toxicity levels for *O. mykiss* are listed by the EPA at 22.19 μg/L for acute exposure and 23.8 μg/L for chronic exposure (Environmental Protection Agency 2007). Normalized EC_50_s for acute exposures, or the number in which half of the fish culture experienced mortality, ranged from 8.10 to 99.97 μg/L, highlighting the variability in Cu bioavailability and therefore toxicity (Environmental Protection Agency 2007). Copper exists in a variety of chemical species within water, with the bioavailable concentrations typically being low, meaning that concentration is not always linked directly to toxicity (Environmental Protection Agency 2007). So, while Cu discharge concentrations of PG&E’s utility vault water study average at 67 μg/L and peak at 791 μg/L, this does not necessarily mean that the biologic community would be impacted. Loading of Cu from urban runoff also comes from a variety of sources (including Cu roofs, roadways, etc.) at concentrations much higher and more frequent than discharges from utility vaults and underground structures (Charters et al. 2016).

Zinc toxicity levels for *O. mykiss* are listed at 689.3 μg/L for acute exposure and a chronic exposure limit is not listed (Environmental Protection Agency 1996b). Even the highest concentration (386 μg/L) utility vault water discharge was below this acute level, meaning that even if the utility vault water was discharged into a small stream, an impact of Zn would not be seen. Utility vault water discharges are short term and intermittent in nature (California State Water Resources Control Board 2014a) so these levels are not representative of chronic exposure.

### Utility vault loads into San Francisco Bay

**Fig. 3:**
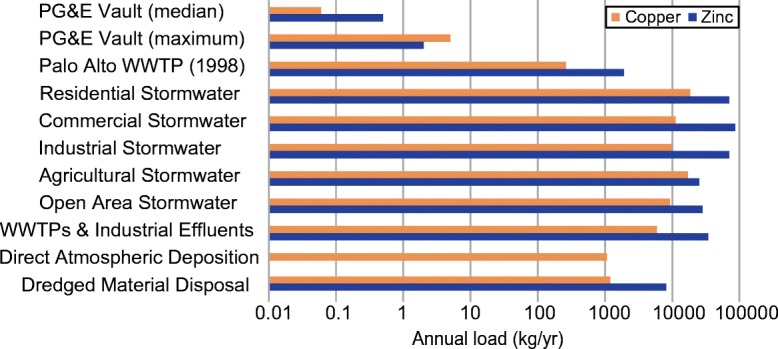
Copper and zinc loads (kg/year) into San Francisco Bay from utility vault water were substantially lower than the Palo Alto Wastewater Treatment Plant (WWTP) (Hornberger et al. 2000), stormwater (e.g., residential, commercial, etc.) (Davis et al. 2000), and multiple other pathways into the Bay (Davis et al. 2000). Note the logarithmic scale on the *x*-axis. The estimated load for WWTPs and industrial effluents includes the largest dischargers into the Bay (14 WWTPs and 6 industrial dischargers). Zn atmospheric deposition was not measured by Davis et al. (2000)

When total volume of PG&E utility vault water discharges into the Bay (only RWQCB Regions 2 and 5 data) during the 2015/2016 monitoring year were combined with median Cu and Zn concentrations from the full PG&E data set, annual loads of Cu and Zn into the Bay were approximately 0.06 kg/year and 0.5 kg/year, respectively (Fig. 3). These results are much lower than the estimated annual loads of 74,000 kg/year of Cu and 320,000 kg/year of Zn to the Bay from all sources, including wastewater treatment plants, industrial effluents, atmospheric deposition, dredging operations and stormwater runoff (Davis et al. 2000). Of these myriad sources, stormwater (including runoff from residential, industrial, commercial, agricultural, and open areas) is by far the largest, accounting for approximately 90% of the total loading of Cu and Zn into the Bay (Davis et al. 2000). A 4-year study of single urban stormwater outfall in Hayward, a city which discharges into the Bay, indicated that Cu and Zn loads were 10 kg/year and 65 kg/year, respectively (McKee and Gilbreath 2015).

When the Cu and Zn utility vault water loads are compared to typical annual discharge loads of a single wastewater treatment plant located in Palo Alto, CA, the average loads from utility vault water discharges are minimal (Fig. 3). Annual discharge loads of Cu and Zn from the Palo Alto Wastewater Treatment Plant were estimated to be 263 kg/year and 1903 kg/year, respectively, in 2000 (Hornberger et al. 2000). By comparison, annual median Bay utility vault water loading for Cu and Zn would comprise less than 0.03% of this wastewater treatment plant’s load for each of these metals. When the maximum detections for Cu and Zn from PG&E’s utility vault data were used (i.e., 791 and 386 μg/L, respectively) in conjunction with the volume and number of discharges into the Bay, utility vault loading comprised 2% of Cu and 0.1% of Zn loading from the Palo Alto Wastewater Treatment Plant. For perspective, there are 46 additional wastewater treatment plants in the San Francisco Bay Area alone (City of Palo Alto 2016).

### Utility vault water loads to state waters

Statewide median annual loads for all of PG&E’s utility vaults (RWQCB Regions 1, 2, 3, and 5 data) into California surface waters are 0.07 kg/year for Cu and 0.6 kg/year for Zn. These loads are only slightly higher than our estimates for PG&E loads into San Francisco Bay alone (regions 2 and 5) of 0.06 kg/year and 0.5 kg/year, respectively. The similarity is driven by discharge patterns; 89% of PG&E’s self-reported discharge (by number of utility vault discharges) occurs in regions 2 and 5 (Pacific Gas and Electric Company 2016a, b, c, d, f). Region 2 is the heavily urbanized San Francisco Bay Area, home to > 7 million people, and utility vaults are most commonly found in urbanized environments. Accordingly, most of the Cu and Zn load from PG&E utility vault waters goes to San Francisco Bay, even though region 2 had the lowest Cu and Zn concentrations among the regions.

When the median Cu and Zn results from PG&E’s utility vault data are multiplied by 74 (i.e., the total number of 2006 NPDES Utility Vault Permit holders) to estimate total loads for utility vault releases from all Dischargers statewide, annual loads are 5 kg/year for Cu and 40 kg/year for Zn. Accordingly, statewide utility vault loads are roughly equivalent to the loads from a small urban watershed. For example, loads from a small (4.17 km^2^) urban watershed that includes Hayward, CA, were relatively low: 10 kg/year for Cu and 65 kg/year for Zn (McKee and Gilbreath 2015). Average annual Cu loads from a medium-sized (330 km^2^) urban watershed (Ballona Creek Watershed), which includes a major portion of Los Angeles, CA, were 250 kg/year for dry weather flow and 1060 kg/year for wet weather flow (McPherson et al. 2005). Loads from a large (835 km^2^) urban watershed (Los Angeles River) were given on a daily basis and reported at 1.036 kg/day for Cu and 2.3 kg/day for Zn (Stein and Tiefenthaler 2005). Thus, the total annual median loading for all utility vaults statewide is roughly equivalent to the loading from a small urban watershed. If the worst-case pollutant load within utility vaults is assumed, the total annual statewide maximum utility vault loads of 400 kg/year for Cu and 200 kg/year for Zn would be roughly equivalent to the annual pollutant load of one medium-sized urban watershed. However, the annual median pollutant load of Cu and Zn from PG&E’s utility vaults alone (0.07 and 0.6 kg/year, respectively) is substantially less than the daily loading from a large urban watershed (1.036 and 2.3 kg/day, respectively) (Pacific Gas and Electric Company 2016a, b, c, d; Stein and Tiefenthaler 2005).

Reported information from the PG&E data set show that 67% of its utility vault water dewatering occurs in the heavily urbanized region 2 (San Francisco Bay Region). Of the 2105 PG&E discharges from the 2015/2016 monitoring year, approximately 7% occurred in region 1, 4% occurred in region 3, and 22% occurred in region 5. The PG&E service area does not cover the southern half of California, where the urbanized Los Angeles area is located. Based on the PG&E dataset, we would expect that the remaining discharges are disproportionately occurring in Los Angeles, but this is an area that needs further investigation. However, the Los Angeles area does not drain into San Francisco Bay, meaning that our estimate is likely representative of the utility vault water loadings to San Francisco Bay, but may need further refinement statewide.

### Challenges of estimating loadings statewide

We made several simplifications to estimate and scale loading data to a statewide level. First, we assumed that all Dischargers are similar to PG&E in terms of volume of discharge and type of contaminants. This simplification is not likely true, but it is difficult to quantify differences between Dischargers. Pipes and wires are common in utility vaults, making metal contamination likely for a variety of different Dischargers. However, unlike PG&E, not all Dischargers have equipment containing oil within their utility vaults.

Not every Discharger under the Utility Vault NPDES Permit will discharge the same volume or frequency as PG&E. We expected other large California utility companies, such as AT&T, Comcast, Verizon, etc., to have discharge patterns similar to PG&E’s. However, their discharge volume and distribution were not quantified for this study. We expected California utility companies smaller than PG&E, such as Sacramento Municipal Utility District, Redding Electric Utility, and Burbank Water and Power, to have far fewer utility vaults and discharges than PG&E, which would make our loadings calculations based on PG&E data an overestimate. To overcome this hurdle and evaluate the full impact of utility vault water loading, we would need additional data. We would need locations and volumes of all of utility vault discharges from all Dischargers. Alternatively, we could potentially scale the PG&E data up to estimate the number of utility vaults statewide by using a geospatial approach to develop a relationship between the number of utility vaults and land use (e.g., industrial, urban, residential, etc.). However, this approach would require us to have geographic coordinates for the utility vault locations, data which are not publicly released.

Only about 75% of the Dischargers from the 2006 NPDES Utility Vault Permit have reapplied for coverage under the 2014 NPDES Utility Vault Permit at the time of this study. Accordingly, our calculated loads could be more than that performed by actual Dischargers (California State Water Resources Control Board 2016). As a whole, since we have utilized the total number of 2006 NPDES Utility Vault Permit holders (more than under the current 2014 permit), we believe that our study provides an over-estimate of the total utility vault loading statewide.

To further refine loadings estimates, we would also like to evaluate the variability of discharge volume. The water year of this study (fall 2015–2016) was the first year that PG&E was required to publicly report utility vault water discharge volume. While water year 2016 was wetter than the critically dry years of 2014 and 2015, it was still relatively dry. Water year 2016 was a below normal water year in the Sacramento Valley and a dry year in the San Joaquin Valley (Anderson 2017). We do not have a good estimate of the variability in utility vault water discharge volume from wet to dry years. However, we expect that greater volume during wet years would increase loads. 

## Conclusion

Overall, pollutant levels and calculated loads indicate that discharges from utility vaults and underground structures do not likely have a “reasonable potential” to cause an exceedance of water quality criteria as long as provisions of the 2014 NPDES Utility Vault Permit are followed. As defined, a “reasonable potential” trigger exists when pollutant concentrations within the discharge are determined to be greater than the background pollutant concentrations (California Environmental Protection Agency 2005).

Utility vault data collected by PG&E indicate that utility vault water contains 21 of the 126 listed priority pollutants within the 2014 NPDES Utility Vault Permit. Of the 21 detected constituents, only two constituents (Cu and Zn) had concentrations above the listed water quality objective levels set within the 2014 NPDES Utility Vault Permit. Contaminant concentrations in utility vault water were generally comparable to concentrations in stormwater, and loads were comparatively small. For example, the median pollutant loads of Cu and Zn from PG&E utility vaults over the course of an entire year (0.07 and 0.6 kg/year, respectively) were less than the pollutant loads from a large urban watershed in Los Angeles (1.036 and 2.3 kg/day, respectively) over the course of a single day. Because stormwater is the likely source of metals in utility vault water and the major source of metal loadings to surface waters, a focus on reducing pollutant load in urban runoff may be more productive than a focus on further controlling utility vault discharges.

Utility vault discharges are typically low in volume and intermittent in nature (California State Water Resources Control Board 2014a). Specifically, PG&E’s total estimated annual discharge from utility vaults was 7000 m^3^. For comparison, annual mean stormwater discharge from a single tributary to the Bay was two orders of magnitude higher, at 720,000 m^3^ (McKee and Gilbreath 2015). As a result, less volume of utility vault water is released when compared to stormwater runoff or municipal wastewater outflow. If the maximum Cu and Zn data results are assumed to be within each and every PG&E utility vault water discharge (i.e., maximum potential impact), total loads for all of PG&E utility vaults into the San Francisco Bay Watershed (regions 2 and 5) are estimated at 5 kg/year for Cu and 2 kg/year for Zn. These loads account for 0.007% of the Cu loading and 0.0009% of the Zn loading to the Bay (Fig. 3).

As a result of the initial findings in this study, it is recommended that the SWRCB continue the SIP exemption in the next iteration of the Utility Vault NPDES Permit. Additionally, it is recommended that data collection and review continue to either support or reject these findings, as this is the first known study and review of utility vault water data and was collected under the first year of the 2-year Characterization Study 1 requirements of the 2014 NPDES Utility Vault Permit. From the data available so far, the loading from utility vaults shows minimal evidence of contributing to surface water pollution, or contributing to an instream exceedance of water quality objectives, as listed within the 2014 NPDES Utility Vault Permit.

## Electronic supplementary material

ESM 1 (PDF 898 MB)
