# Supplementary material for: Assessment of the contribution of utility vault water to surface water pollution
Source: Environ Monit Assess. 2019 Jun 26;191(7):467. doi: 10.1007/s10661-019-7585-y (PMC6594985; doi:10.1007/s10661-019-7585-y)
Supplement: Supplementary file 1 — (PDF 898 MB) [file 10661_2019_7585_MOESM1_ESM.pdf]

Jeremy T. Laurin[a,b] and Allison C. Luengen[a *]
a. Environmental Sciences Department, University of San Francisco, 2130 Fulton Street, San Francisco, CA, 94117, USA
b. Environmental Management, Pacific Gas & Electric Company, 3401 Crow Canyon Road, San Ramon, CA 94583, USA
* corresponding author, email: aluengen@usfca.edu

Online Resource Caption:

Analytical Results for PG&E Utility Vault Samples collected by PG&E as a provision of their permit coverage under WQO-2014-0174 (the 2014 NPDES Vault Permit) and reported in their 2015/2016 Annual Monitoring Reports, within Appendix C (Pacific Gas and Electric Company 2016a, 2016b, 2016c, 2016d). The data were collected in the 2015/2016 monitoring year (November – May) and represent the first year of data collection for Characterization Study 1 as required by the 2014 NPDES Vault Permit. Quality control data are available in the reports mentioned above. Note that due to a broken bottle, our calculations used the data from the "B" sample for the Region 5 V5-R5 vault, available with quality control data.

Jeremy T. Laurin[a,b] and Allison C. Luengen[a *]
a. Environmental Sciences Department, University of San Francisco, 2130 Fulton Street, San Francisco, CA, 94117, USA
b. Environmental Management, Pacific Gas & Electric Company, 3401 Crow Canyon Road, San Ramon, CA 94583, USA
* corresponding author, email: aluengen@usfca.edu

Online Resource - Analytical Results for PG&E Utility Vault Water Samples

				Gen Min	Dioxin	Gen Min	Gen Min	Gen Min	Metals	Metals	Metals	Metals	Metals	Metals	Metals	Metals	Metals	Metals	Metals	Metals	Metals	Metals	OG	PAH	PAH	PAH	PAH
Region	Vault Location ID	Sample ID	Sample Date	pH - field measurement (Standard Units)	2,3,7,8-TCDD (pg/l)	Cyanide, Total (mg/L)	Hardness, Total (as CaCO3) (mg/L)	Total Suspended Solids (mg/L)	Antimony (mg/L)	Arsenic (mg/L)	Beryllium (mg/L)	Cadmium (mg/L)	Chromium-Total (mg/L)	Copper (mg/L)	Hexavalent Chromium (EPA 7199) (µg/L)	Lead (mg/L)	Mercury (ng/L)	Nickel (mg/L)	Selenium (mg/L)	Silver (mg/L)	Thallium (mg/L)	Zinc (mg/L)	HEM: Oil and Grease (mg/L)	1-Methylnaphthalene (µg/L)	2-Methylnaphthalene (µg/L)	Acenaphthene (µg/L)	Acenaphthylene (µg/L)
Water Quality Criteria (µg/L)				--	0.000000014	22	--	--	4300	340	--	Hardness Based	Hardness Based	Hardness Based	16	Hardness Based	0.051	Hardness Based	Hardness Based	Hardness Based	6.3	Hardness Based	--	--	--	2700	--
Water Quality Criteria (mg/L)				--	1.4E-11	0.022	--	--	4.3	0.34	--	Hardness Based	Hardness Based	Hardness Based	0.016	Hardness Based	0.000051	Hardness Based	Hardness Based	Hardness Based	0.0063	Hardness Based	--	--	--	2.7	--
Minimum Level (µg/L)				--	Special	5	--	--	0.5	1	0.5	0.25	0.5	0.5	5	0.5	0.2	1	1	0.25	1	1	--	--	--	0.5	0.2
Region 1																											
R1	V1-R1	V1-R1-EFF-A	1/7/2016	7.96	<0.354	<0.0070	130	<0.83	0.000788 J	<0.000386	<0.000290	<0.000128	0.000925 J	0.791	0.12 BV,J,BU	0.00243	4.27	0.00160	<0.000168	<0.000111	<0.000101	0.227	<0.80	<0.074	<0.075	<1.4	<1.4
R1	V1-R1	V1-R1-EFF-A	4/5/2016	--	--	--	--	--	--	--	--	--	--	--	--	--	--	--	--	--	--	--	--	--	--	--	--
R1	V2-R1	V2-R1-EFF-A	1/7/2016	7.72	<0.326	<0.0070	350	<0.83	0.00107	0.00118	<0.000290	0.000182 J	0.000545 J	0.0158	<0.067 BV,BU	0.000409 J	2.85	0.00250	<0.000168	<0.000111	<0.000101	0.0964	0.96 J	<0.074	<0.075	<1.4	<1.4
R1	V2-R1	V2-R1-EFF-A	4/5/2016	--	--	--	--	--	--	--	--	--	--	--	--	--	--	--	--	--	--	--	--	--	--	--	--
R1	V3-R1	V3-R1-EFF-A	1/7/2016	7.92	<0.357	<0.0070	87	<0.83	0.000330 J	<0.000386	<0.000290	<0.000128	0.000793 J	0.00510	<0.067	0.000332 J	1.51	0.00156	<0.000168	<0.000111	<0.000101	0.386	<0.80	<0.074	<0.075	<1.4	<1.4
R1	V3-R1	V3-R1-EFF-A	4/5/2016	--	--	--	--	--	--	--	--	--	--	--	--	--	--	--	--	--	--	--	--	--	--	--	--
R1	V4-R1	V4-R1-EFF-A	1/7/2016	8.16	<0.286	<0.0070	220	<0.83	0.00173	0.00350	<0.000290	0.000181 J	0.000483 J	0.00956	0.19 J	0.000264 J	10.4	0.00495	0.000264 J	<0.000111	<0.000101	0.168	<0.80	<0.074	<0.075	<1.4	<1.4
R1	V4-R1	V4-R1-EFF-A	4/5/2016	--	--	--	--	--	--	--	--	--	--	--	--	--	--	--	--	--	--	--	--	--	--	--	--
R1	V5-R1	V5-R1-EFF-A	1/11/2016	7.25	<0.468	<0.0070	47	10	<0.0000995	<0.000386	<0.000290	<0.000128	0.00127	0.00444	0.11 BV,J,BU	0.000462 J	2.78	0.00113	<0.000168	<0.000111	<0.000101	0.0183	1.5	<0.074	<0.075	<1.4	<1.4
R1	V5-R1	V5-R1-EFF-A	3/28/2016	--	--	--	--	--	--	--	--	--	--	--	--	--	--	--	--	--	--	--	--	--	--	--	--
Region 2																											
R2	V1-R2	V1-R2-EFF	12/1/2015	7.35	<0.611	<0.0070	350	<0.83	0.000818 J	<0.000386	<0.000290	<0.000128	0.000490 J	0.00325	<0.067	0.00278	1.15	0.00456	<0.000168	<0.000111	<0.000101	0.0740	<0.80	<0.076	<0.076	<1.4	<1.4
R2	V1-R2	V1-R2-EFF	3/22/2016	--	--	--	--	--	--	--	--	--	--	--	--	--	--	--	--	--	--	--	--	--	--	--	--
R2	V2-R2	V2-R2-EFF	12/1/2015	7.88	<0.770	<0.0070	330	<0.83	0.000636 J	0.00116	<0.000290	<0.000128	0.000424 J	0.00344	<0.067	0.00785	0.702	0.00757	0.000228 J	<0.000111	<0.000101	0.0631	<0.80	<0.076	<0.076	<1.3	<1.4
R2	V2-R2	V2-R2-EFF	3/22/2016	--	--	--	--	--	--	--	--	--	--	--	--	--	--	--	--	--	--	--	--	--	--	--	--
R2	V3-R2	V3-R2-EFF	12/1/2015	8.01	<0.509	<0.0070	170	<0.83	0.000660 J	0.00192	<0.000290	<0.000128	0.000467 J	0.00966	<0.067	0.000825 J	0.746	0.00394	0.000312 J	<0.000111	<0.000101	0.0995	<0.80	<0.076	<0.076	<1.4	<1.4
R2	V3-R2	V3-R2-EFF	3/22/2016	--	--	--	--	--	--	--	--	--	--	--	--	--	--	--	--	--	--	--	--	--	--	--	--
R2	V4-R2	V4-R2-EFF	12/15/2015	7.84	<0.907	<0.0070	190	<0.83	0.0111	0.00218	<0.000290	0.000203 J	0.000551 J	0.0205	0.27 J	0.000219 J	3.41	0.00159	0.000504 J	<0.000111	<0.000101	0.0342	<0.80	<0.076	<0.076	<1.4	<1.4
R2	V4-R2	V4-R2-EFF	3/30/2016	--	--	--	--	--	--	--	--	--	--	--	--	--	--	--	--	--	--	--	--	--	--	--	--
R2	V5-R2	V5-R2-EFF	12/15/2015	7.79	<0.804	<0.0070	220	<0.83	0.00236	0.000916 J	<0.000290	0.000425 J	0.00313	0.0104	2.5	0.00127	4.93	0.00181	0.00266	<0.000111	<0.000101	0.0203	1.1	<0.074	<0.075	<1.4	<1.4
R2	V5-R2	V5-R2-EFF	3/30/2016	--	--	--	--	--	--	--	--	--	--	--	--	--	--	--	--	--	--	--	--	--	--	--	--
Region 3																											
R3	V1-R3	V1-R3-EFF	12/2/2015	7.51	<0.425	<0.0070	270	<0.83	0.000591 J	<0.000386	<0.000290	0.000159 J	0.00135	0.0880	0.35 BV,J,BU	0.000125 J	0.510	0.00321	0.000524 J	<0.000111	<0.000101	0.307	<0.80	<0.074	<0.075	<1.4	<1.4
R3	V1-R3	V1-R3-EFF	3/17/2016	--	--	--	--	--	--	--	--	--	--	--	--	--	--	--	--	--	--	--	--	--	--	--	--
R3	V2-R3	V2-R3-EFF	12/2/2015	8.02	<0.512	<0.0070	400	1.7	0.00560	0.0226	<0.000290	<0.000128	0.000915 J	0.00176	<0.067 BU	0.000189 J	<0.113	0.00531	0.000185 J	<0.000111	<0.000101	0.106	<0.80	<0.074	<0.075	<1.3	<1.4
R3	V2-R3	V2-R3-EFF	3/17/2016	--	--	--	--	--	--	--	--	--	--	--	--	--	--	--	--	--	--	--	--	--	--	--	--
R3	V3-R3	V3-R3-EFF	12/2/2015	8.4	<0.387	<0.0070	320	<0.83	0.00186	0.00310	<0.000290	<0.000128	0.00146	0.0135	0.80 J	<0.0000898	0.225 J	0.00192	0.000297 J	<0.000111	<0.000101	0.0201	<0.80	<0.073	<0.073	<1.4	<1.4
R3	V3-R3	V3-R3-EFF	3/17/2016	--	--	--	--	--	--	--	--	--	--	--	--	--	--	--	--	--	--	--	--	--	--	--	--
R3	V4-R3	V4-R3-EFF	12/2/2015	8.28	<0.457	<0.0070	260	<0.83	0.00653	0.000700 J	<0.000290	<0.000128	0.000797 J	0.00819	<0.067	<0.0000898	<0.113	0.00459	<0.000168	<0.000111	<0.000101	0.0168	<0.80	<0.074	<0.075	<1.4	<1.4
R3	V4-R3	V4-R3-EFF	3/17/2016	--	--	--	--	--	--	--	--	--	--	--	--	--	--	--	--	--	--	--	--	--	--	--	--
R3	V5-R3	V5-R3-EFF	12/3/2015	8.27	<0.472	<0.0070	330	<0.83	0.0253	0.00138	<0.000290	<0.000128	0.00207	0.116	<1.3 BV,BU	0.00173	0.952	0.00174	0.00607	<0.000111	<0.000101	0.0262	<0.80	<0.073	<0.073	<1.3	<1.4
R3	V5-R3	V5-R3-EFF	3/16/2016	--	--	--	--	--	--	--	--	--	--	--	--	--	--	--	--	--	--	--	--	--	--	--	--
Region 5																											
R5	V1-R5	V1-R5-A	11/23/2015	8.27	<0.630	<0.0070	350	<0.83	0.000351 J	<0.000386	<0.000290	<0.000128	0.00154	0.00636	0.75 J	0.0000927 J	0.919	0.00170	0.000547 J	<0.000111	<0.000101	0.0991	<0.80	<0.073	<0.073	<1.3	<1.4
R5	V1-R5	V1-R5-A	3/21/2016	--	--	--	--	--	--	--	--	--	--	--	--	--	--	--	--	--	--	--	--	--	--	--	--
R5	V2-R5	V2-R5-A	11/23/2015	8.10	<0.393	<0.0070	190	<0.83	0.000637 J	0.00160	<0.000290	<0.000128	0.00273	0.0510	1.9	0.000175 J	3.23	0.00275	0.000694 J	<0.000111	<0.000101	0.166	<0.80	<0.074	<0.074	<1.3	<1.4
R5	V2-R5	V2-R5-A	3/21/2016	--	--	--	--	--	--	--	--	--	--	--	--	--	--	--	--	--	--	--	--	--	--	--	--
R5	V3-R5	V3-R5-EFF-A	11/23/2015	7.74	<0.346	<0.0070	280	<0.83	0.000293 J	0.00463	<0.000290	<0.000128	0.00263	0.000284 J	2.1	0.000259 J	1.68	0.00144	0.000468 J	<0.000111	<0.000101	0.0892	<0.80	<0.073	<0.073	<1.3	<1.4
R5	V3-R5	V3-R5-EFF-A	3/21/2016	--	--	--	--	--	--	--	--	--	--	--	--	--	--	--	--	--	--	--	--	--	--	--	--
R5	V4-R5	V4-R5-A	11/23/2015	8.46	<0.377	<0.0070	140	<0.83	0.00647	0.0890	<0.000290	<0.000128	0.00262	0.0124	1.9	0.000680 J	3.43	0.00101	0.000841 J	<0.000111	<0.000101	0.0157	<0.80	<0.072	<0.073	<1.3	<1.4
R5	V4-R5	V4-R5-A	3/21/2016	--	--	--	--	--	--	--	--	--	--	--	--	--	--	--	--	--	--	--	--	--	--	--	--
R5	V5-R5	V5-R5-A	11/23/2015	8.47	<0.375	<0.0070	47	<0.83	0.0935	0.0771	<0.000290	<0.000128	0.0119	0.00716	10	0.000334 J	<45.3	0.000910 J	0.00728	<0.000111	<0.000101	0.0428	<0.80	<0.073	<0.073	<1.3	<1.4
R5	V5-R5	V5-R5-A	3/21/2016	--	--	--	--	--	--	--	--	--	--	--	--	--	--	--	--	--	--	--	--	--	--	--	--

Jeremy T. Laurin[a,b] and Allison C. Luengen[a *]
a. Environmental Sciences Department, University of San Francisco, 2130 Fulton Street, San Francisco, CA, 94117, USA
b. Environmental Management, Pacific Gas & Electric Company, 3401 Crow Canyon Road, San Ramon, CA 94583, USA
* corresponding author, email: aluengen@usfca.edu

Online Resource - Analytical Results for PG&E Utility Vault Water Samples

Region	Vault Location ID	Sample ID	Sample Date	Anthracene (µg/L)	Benzo(a)anthracene (µg/L)	Benzo(a)pyrene (µg/L)	Benzo(b)fluoranthene (µg/L)	Benzo(g,h,i)perylene (µg/L)	Benzo(k)fluoranthene (µg/L)	Chrysene (µg/L)	Dibenz(a,h)anthracene (µg/L)	Fluoranthene (µg/L)	Fluorene (µg/L)	Indeno(1,2,3-c,d)pyrene (µg/L)	Naphthalene (8270) (µg/L)	Naphthalene (8270SIM) (µg/L)	Phenanthrene (µg/L)	Pyrene (µg/L)	Aroclor 1016 (µg/L)	Aroclor 1221 (µg/L)	Aroclor 1232 (µg/L)	Aroclor 1242 (µg/L)	Aroclor 1248 (µg/L)	Aroclor 1254 (µg/L)	Aroclor 1260 (µg/L)
				PAH	PAH	PAH	PAH	PAH	PAH	PAH	PAH	PAH	PAH	PAH	PAH	PAH	PAH	PAH	PCB	PCB	PCB	PCB	PCB	PCB	PCB
Water Quality Criteria (µg/L)				110000	0.049	0.049	0.049	--	0.049	0.049	0.049	370	14000	0.049	--	--	--	11000	0.00017 (Sum of PCBs)						
Water Quality Criteria (mg/L)				110	0.000049	0.000049	0.000049	--	0.000049	0.000049	0.000049	0.37	14	0.000049	--	--	--	11	0.00000017 (Sum of PCBs)						
Minimum Level (µg/L)				2	5	2	10	0.1	2	5	0.1	0.05	0.1	0.05	0.2	0.2	0.05	0.05	0.5	0.5	0.5	0.5	0.5	0.5	0.5
Region 1																									
R1	V1-R1	V1-R1-EFF-A	1/7/2016	<1.5	<2.3	<1.2	<1.1	<1.2	<1.6	<1.4	<1.2	<1.5	<1.3	<1.0	<1.4	<0.073	<1.4	<1.4	<0.14	<0.14	<0.12	<0.061	<0.098	<0.11	<0.13
R1	V1-R1	V1-R1-EFF-A	4/5/2016	--	--	--	--	--	--	--	<0.0047	--	--	<0.0051	--	--	--	--	--	--	--	--	--	--	--
R1	V2-R1	V2-R1-EFF-A	1/7/2016	<1.5	<2.3	<1.2	<1.1	<1.2	<1.6	<1.4	<1.2	<1.5	<1.3	<1.1	<1.4	<0.073	<1.4	<1.5	<0.14	<0.14	<0.12	<0.061	<0.098	<0.11	<0.13
R1	V2-R1	V2-R1-EFF-A	4/5/2016	--	--	--	--	--	--	--	<0.0047	--	--	<0.0051	--	--	--	--	--	--	--	--	--	--	--
R1	V3-R1	V3-R1-EFF-A	1/7/2016	<1.5	<2.3	<1.2	<1.1	<1.2	<1.6	<1.4	<1.2	<1.5	<1.3	<1.0	<1.4	<0.073	<1.4	<1.4	<0.14	<0.14	<0.12	<0.061	<0.098	<0.11	<0.13
R1	V3-R1	V3-R1-EFF-A	4/5/2016	--	--	--	--	--	--	--	<0.0047	--	--	<0.0051	--	--	--	--	--	--	--	--	--	--	--
R1	V4-R1	V4-R1-EFF-A	1/7/2016	<1.5	<2.3	<1.2	<1.1	<1.2	<1.6	<1.4	<1.2	<1.5	<1.3	<1.0	<1.4	<0.073	<1.4	<1.4	<0.14	<0.14	<0.12	<0.061	<0.098	<0.11	<0.13
R1	V4-R1	V4-R1-EFF-A	4/5/2016	--	--	--	--	--	--	--	<0.0047	--	--	<0.0051	--	--	--	--	--	--	--	--	--	--	--
R1	V5-R1	V5-R1-EFF-A	1/11/2016	<1.5	<2.3	<1.2	<1.1	<1.2	<1.6	<1.4	<1.2	<1.5	<1.3	<1.0	<1.4	<0.073	<1.4	<1.4	<0.14	<0.14	<0.12	<0.060	<0.097	<0.11	<0.13
R1	V5-R1	V5-R1-EFF-A	3/28/2016	--	--	--	--	--	--	--	<0.0046	--	--	<0.0050	--	--	--	--	--	--	--	--	--	--	--
Region 2																									
R2	V1-R2	V1-R2-EFF	12/1/2015	<1.5	<2.3	<1.2	<1.1	<1.2	<1.6	<1.4	<1.2	<1.5	<1.3	<1.1	<1.4	<0.075	<1.4	<1.5	<0.15	<0.14	<0.12	<0.063	<0.10	<0.11	<0.13
R2	V1-R2	V1-R2-EFF	3/22/2016	--	--	--	--	--	--	--	<0.0046	--	--	<0.0050	--	--	--	--	--	--	--	--	--	--	--
R2	V2-R2	V2-R2-EFF	12/1/2015	<1.5	<2.2	<1.2	<1.1	<1.2	<1.6	<1.4	<1.2	<1.5	<1.3	<1.0	<1.4	<0.075	<1.4	<1.4	<0.15	<0.14	<0.12	<0.063	<0.10	<0.11	<0.13
R2	V2-R2	V2-R2-EFF	3/22/2016	--	--	--	--	--	--	--	<0.0046	--	--	<0.0050	--	--	--	--	--	--	--	--	--	--	--
R2	V3-R2	V3-R2-EFF	12/1/2015	<1.5	<2.3	<1.2	<1.1	<1.2	<1.6	<1.4	<1.2	<1.5	<1.3	<1.1	<1.4	<0.075	<1.4	<1.5	<0.14	<0.14	<0.12	<0.061	<0.099	<0.11	<0.13
R2	V3-R2	V3-R2-EFF	3/22/2016	--	--	--	--	--	--	--	<0.0046	--	--	<0.0050	--	--	--	--	--	--	--	--	--	--	--
R2	V4-R2	V4-R2-EFF	12/15/2015	<1.5	<2.3	<1.2	<1.1	<1.2	<1.6	<1.4	<1.2	<1.5	<1.3	<1.1	<1.4	<0.075	<1.4	<1.5	<0.15	<0.14	<0.12	<0.063	<0.10	<0.11	<0.13
R2	V4-R2	V4-R2-EFF	3/30/2016	--	--	--	--	--	--	--	<0.0046	--	--	<0.0050	--	--	--	--	--	--	--	--	--	--	--
R2	V5-R2	V5-R2-EFF	12/15/2015	<1.5	<2.3	<1.2	<1.1	<1.2	<1.6	<1.4	<1.2	<1.5	<1.3	<1.1	<1.4	<0.073	<1.4	<1.5	<0.14	<0.14	<0.12	<0.061	<0.099	<0.11	<0.13
R2	V5-R2	V5-R2-EFF	3/30/2016	--	--	--	--	--	--	--	<0.0046	--	--	<0.0050	--	--	--	--	--	--	--	--	--	--	--
Region 3																									
R3	V1-R3	V1-R3-EFF	12/2/2015	<1.5	<2.3	<1.2	<1.1	<1.2	<1.6	<1.4	<1.2	<1.5	<1.3	<1.0	<1.4	<0.073	<1.4	<1.4	<0.14	<0.14	<0.12	<0.060	<0.097	<0.11	<0.13
R3	V1-R3	V1-R3-EFF	3/17/2016	--	--	--	--	--	--	--	<0.0046	--	--	<0.0050	--	--	--	--	--	--	--	--	--	--	--
R3	V2-R3	V2-R3-EFF	12/2/2015	<1.5	<2.2	<1.2	<1.1	<1.2	<1.6	<1.4	<1.2	<1.5	<1.3	<1.0	<1.4	<0.073	<1.4	<1.4	<0.14	<0.14	<0.12	<0.060	<0.097	<0.11	<0.13
R3	V2-R3	V2-R3-EFF	3/17/2016	--	--	--	--	--	--	--	<0.0046	--	--	<0.0050	--	--	--	--	--	--	--	--	--	--	--
R3	V3-R3	V3-R3-EFF	12/2/2015	<1.5	<2.3	<1.2	<1.1	<1.2	<1.6	<1.4	<1.2	<1.5	<1.3	<1.1	<1.4	<0.072	<1.4	<1.5	<0.14	<0.14	<0.12	<0.060	<0.097	<0.11	<0.13
R3	V3-R3	V3-R3-EFF	3/17/2016	--	--	--	--	--	--	--	<0.0046	--	--	<0.0050	--	--	--	--	--	--	--	--	--	--	--
R3	V4-R3	V4-R3-EFF	12/2/2015	<1.5	<2.3	<1.2	<1.1	<1.2	<1.6	<1.4	<1.2	<1.5	<1.3	<1.1	<1.4	<0.073	<1.4	<1.5	<0.14	<0.14	<0.12	<0.060	<0.097	<0.11	<0.13
R3	V4-R3	V4-R3-EFF	3/17/2016	--	--	--	--	--	--	--	<0.0046	--	--	<0.0050	--	--	--	--	--	--	--	--	--	--	--
R3	V5-R3	V5-R3-EFF	12/3/2015	<1.5	<2.2	<1.2	<1.1	<1.2	<1.6	<1.4	<1.2	<1.5	<1.3	<1.0	<1.4	<0.072	<1.4	<1.4	<0.14	<0.14	<0.12	<0.060	<0.097	<0.11	<0.13
R3	V5-R3	V5-R3-EFF	3/16/2016	--	--	--	--	--	--	--	<0.0046	--	--	<0.0050	--	--	--	--	--	--	--	--	--	--	--
Region 5																									
R5	V1-R5	V1-R5-A	11/23/2015	<1.5	<2.2	<1.2	<1.1	<1.2	<1.6	<1.4	<1.2	<1.5	<1.3	<1.0	<1.4	<0.072	<1.4	<1.4	<0.14	<0.14	<0.12	<0.060	<0.097	<0.11	<0.13
R5	V1-R5	V1-R5-A	3/21/2016	--	--	--	--	--	--	--	<0.0046	--	--	<0.0050	--	--	--	--	--	--	--	--	--	--	--
R5	V2-R5	V2-R5-A	11/23/2015	<1.5	<2.2	<1.2	<1.1	<1.2	<1.6	<1.4	<1.2	<1.5	<1.3	<1.0	<1.4	<0.073	<1.4	<1.4	<0.14	<0.14	<0.12	<0.060	<0.097	<0.11	<0.13
R5	V2-R5	V2-R5-A	3/21/2016	--	--	--	--	--	--	--	<0.0046	--	--	<0.0050	--	--	--	--	--	--	--	--	--	--	--
R5	V3-R5	V3-R5-EFF-A	11/23/2015	<1.5	<2.2	<1.2	<1.1	<1.2	<1.6	<1.4	<1.2	<1.5	<1.3	<1.0	<1.4	<0.072	<1.4	<1.4	<0.14	<0.14	<0.12	<0.060	<0.097	<0.11	<0.13
R5	V3-R5	V3-R5-EFF-A	3/21/2016	--	--	--	--	--	--	--	<0.0046	--	--	<0.0050	--	--	--	--	--	--	--	--	--	--	--
R5	V4-R5	V4-R5-A	11/23/2015	<1.5	<2.2	<1.2	<1.1	<1.2	<1.6	<1.4	<1.2	<1.5	<1.3	<1.0	<1.4	<0.071	<1.4	<1.4	<0.14	<0.14	<0.12	<0.060	<0.097	<0.11	<0.13
R5	V4-R5	V4-R5-A	3/21/2016	--	--	--	--	--	--	--	<0.0046	--	--	<0.0050	--	--	--	--	--	--	--	--	--	--	--
R5	V5-R5	V5-R5-A	11/23/2015	<1.5	<2.2	<1.2	<1.1	<1.2	<1.6	<1.4	<1.2	<1.5	<1.3	<1.0	<1.4	<0.072	<1.4	<1.4	<0.14	<0.14	<0.12	<0.060	<0.097	<0.11	<0.13
R5	V5-R5	V5-R5-A	3/21/2016	--	--	--	--	--	--	--	<0.0046	--	--	<0.0050	--	--	--	--	--	--	--	--	--	--	--

Jeremy T. Laurin[a,b] and Allison C. Luengen[a*]
a. Environmental Sciences Department, University of San Francisco, 2130 Fulton Street, San Francisco, CA, 94117, USA
b. Environmental Management, Pacific Gas & Electric Company, 3401 Crow Canyon Road, San Ramon, CA 94583, USA
* corresponding author, email: aluengen@usfca.edu

Online Resource - Analytical Results for PG&E Utility Vault Water Samples

				Pest	Pest	Pest	Pest	Pest	Pest	Pest	Pest	Pest	Pest	Pest	Pest	Pest	Pest	Pest	Pest	Pest	Pest	Pest	SVOC	SVOC	SVOC	SVOC	SVOC	SVOC	SVOC	SVOC	SVOC	SVOC	SVOC	SVOC	SVOC	SVOC	SVOC
Region	Vault Location ID	Sample ID	Sample Date	4,4-DDD (µg/L)	4,4-DDE (µg/L)	4,4-DDT (µg/L)	Aldrin (µg/L)	alpha-BHC (µg/L)	alpha-Endosulfan (µg/L)	beta-BHC (µg/L)	beta-Endosulfan (µg/L)	Chlordane (µg/L)	delta-BHC (µg/L)	Dieldrin (µg/L)	Endosulfan sulfate (µg/L)	Endrin (µg/L)	Endrin aldehyde (µg/L)	gamma-BHC (Lindane) (µg/L)	Heptachlor (µg/L)	Heptachlor epoxide (µg/L)	Methoxychlor (µg/L)	Toxaphene (µg/L)	1,2-Diphenylhydrazine (µg/L)	2,4,6-Trichlorophenol (µg/L)	2,4-Dichlorophenol (µg/L)	2,4-Dimethylphenol (µg/L)	2,4-Dinitrophenol (µg/L)	2,4-Dinitrotoluene (µg/L)	2,6-Dinitrotoluene (µg/L)	2-Chloronaphthalene (µg/L)	2-Chlorophenol (µg/L)	2-Nitrophenol (µg/L)	3,3-Dichlorobenzidine (µg/L)	4,6-Dinitro-2-methylphenol (µg/L)	4-Bromophenyl phenyl ether (µg/L)	4-Chloro-3-methylphenol (µg/L)	4-Chlorophenyl phenyl ether (µg/L)
			Water Quality Criteria (µg/L)	0.00084	0.00059	0.00059	0.00014	0.013	0.22	0.046	0.22	0.00059	--	0.00014	110	0.81	0.76	0.063	0.00021	0.00011	--	0.00075	0.54	6.5	790	2300	14000	9.1	--	4300	400	--	0.077	765	--	--	--
			Water Quality Criteria (mg/L)	0.00000084	0.00000059	0.00000059	0.00000014	0.000013	0.00022	0.000046	0.00022	0.00000059	--	0.00000014	0.11	0.00081	0.00076	0.000063	0.00000021	0.00000011	--	0.00000075	0.00054	0.0065	0.79	2.3	14	0.0091	--	4.3	0.4	--	0.000077	0.765	--	--	--
			Minimum Level (µg/L)	0.05	0.05	0.01	0.005	0.01	0.02	0.005	0.01	0.1	0.005	0.01	0.05	0.01	0.01	0.02	0.01	0.01	--	0.5	1	10	1	1	5	5	5	10	2	10	5	5	5	1	5
Region 1																																					
R1	V1-R1	V1-R1-EFF-A	1/7/2016	<0.013	<0.013	<0.013	<0.013	<0.014	<0.014	<0.015	<0.013	<0.16	<0.014	<0.014	<0.014	<0.015	<0.013	<0.015	<0.013	<0.012	<0.012	<0.29	<0.41	<1.2	<1.2	<1.2	<6.5	<1.1	<1.1	<1.3	<1.1	<1.3	<1.3	<6.9	<1.3	<1.2	<1.3
R1	V1-R1	V1-R1-EFF-A	4/5/2016	--	--	<0.00050	<0.00050	<0.00050	--	--	--	<0.0025	--	<0.00050	--	--	--	--	<0.00050	<0.00050	--	--	--	--	--	--	--	--	--	--	--	--	--	--	--	--	--
R1	V2-R1	V2-R1-EFF-A	1/7/2016	<0.013	<0.013	<0.013	<0.013	<0.014	<0.014	<0.015	<0.013	<0.16	<0.014	<0.014	<0.014	<0.015	<0.013	<0.015	<0.013	<0.012	<0.012	<0.29	<0.42	<1.2	<1.2	<1.2	<6.6	<1.1	<1.2	<1.4	<1.1	<1.3	<1.3	<7.0	<1.3	<1.2	<1.3
R1	V2-R1	V2-R1-EFF-A	4/5/2016	--	--	<0.00050	<0.00050	<0.00050	--	--	--	<0.0025	--	<0.00050	--	--	--	--	<0.00050	<0.00050	--	--	--	--	--	--	--	--	--	--	--	--	--	--	--	--	--
R1	V3-R1	V3-R1-EFF-A	1/7/2016	<0.013	<0.013	<0.013	<0.013	<0.014	<0.014	<0.015	<0.013	<0.16	<0.014	<0.014	<0.014	<0.015	<0.013	<0.015	<0.013	<0.012	<0.012	<0.29	<0.41	<1.2	<1.2	<1.2	<6.5	<1.1	<1.1	<1.3	<1.1	<1.3	<1.3	<6.9	<1.3	<1.2	<1.3
R1	V3-R1	V3-R1-EFF-A	4/5/2016	--	--	<0.00050	<0.00050	<0.00050	--	--	--	<0.0025	--	<0.00050	--	--	--	--	<0.00050	<0.00050	--	--	--	--	--	--	--	--	--	--	--	--	--	--	--	--	--
R1	V4-R1	V4-R1-EFF-A	1/7/2016	<0.013	<0.013	<0.013	<0.013	<0.014	<0.014	<0.015	<0.013	<0.16	<0.014	<0.014	<0.014	<0.015	<0.013	<0.015	<0.013	<0.012	<0.012	<0.29	<0.41	<1.2	<1.2	<1.2	<6.5	<1.1	<1.1	<1.3	<1.1	<1.3	<1.3	<6.9	<1.3	<1.2	<1.3
R1	V4-R1	V4-R1-EFF-A	4/5/2016	--	--	<0.00050	<0.00050	<0.00050	--	--	--	<0.0025	--	<0.00050	--	--	--	--	<0.00050	<0.00050	--	--	--	--	--	--	--	--	--	--	--	--	--	--	--	--	--
R1	V5-R1	V5-R1-EFF-A	1/11/2016	<0.013	<0.013	<0.013	<0.013	<0.013	<0.013	<0.014	<0.013	<0.16	<0.014	<0.014	<0.014	<0.015	<0.013	<0.014	<0.013	<0.012	<0.012	<0.28	<0.41	<1.2	<1.2	<1.2	<6.5	<1.1	<1.1	<1.3	<1.1	<1.3	<1.3	<6.9	<1.3	<1.2	<1.3
R1	V5-R1	V5-R1-EFF-A	3/28/2016	--	--	<0.00050	<0.00050	<0.00050	--	--	--	<0.0025	--	<0.00050	--	--	--	--	<0.00050	<0.00050	--	--	--	--	--	--	--	--	--	--	--	--	--	--	--	--	--
Region 2																																					
R2	V1-R2	V1-R2-EFF	12/1/2015	<0.014	<0.013	<0.013	<0.013	<0.014	<0.014	<0.015	<0.014	<0.17	<0.014	<0.014	<0.015	<0.015	<0.013	<0.015	<0.013	<0.013	<0.013	<0.30	<0.42	<1.2	<1.2	<1.2	<6.6	<1.1	<1.2	<1.4	<1.1	<1.3	<1.3	<7.0	<1.3	<1.2	<1.3
R2	V1-R2	V1-R2-EFF	3/22/2016	--	--	<0.00050	<0.00050	<0.00050	--	--	--	<0.0025	--	<0.00050	--	--	--	--	<0.00050	<0.00050	--	--	--	--	--	--	--	--	--	--	--	--	--	--	--	--	--
R2	V2-R2	V2-R2-EFF	12/1/2015	<0.014	<0.013	<0.013	<0.013	<0.014	<0.014	<0.015	<0.014	<0.17	<0.014	<0.014	<0.015	<0.015	<0.013	<0.015	<0.013	<0.013	<0.013	<0.30	<0.41	<1.2	<1.2	<1.2	<6.5	<1.1	<1.1	<1.3	<1.1	<1.2	<1.2	<6.8	<1.3	<1.1	<1.3
R2	V2-R2	V2-R2-EFF	3/22/2016	--	--	<0.00050	<0.00050	<0.00050	--	--	--	<0.0025	--	<0.00050	--	--	--	--	<0.00050	<0.00050	--	--	--	--	--	--	--	--	--	--	--	--	--	--	--	--	--
R2	V3-R2	V3-R2-EFF	12/1/2015	<0.013	<0.013	<0.013	<0.013	<0.014	<0.014	<0.015	<0.013	<0.16	<0.014	<0.014	<0.014	<0.015	<0.013	<0.015	<0.013	<0.012	<0.012	<0.29	<0.42	<1.2	<1.2	<1.2	<6.6	<1.1	<1.2	<1.4	<1.1	<1.3	<1.3	<7.0	<1.3	<1.2	<1.3
R2	V3-R2	V3-R2-EFF	3/22/2016	--	--	<0.00050	<0.00050	<0.00050	--	--	--	<0.0025	--	<0.00050	--	--	--	--	<0.00050	<0.00050	--	--	--	--	--	--	--	--	--	--	--	--	--	--	--	--	--
R2	V4-R2	V4-R2-EFF	12/15/2015	<0.014	<0.013	<0.013	<0.013	<0.014	<0.014	<0.015	<0.014	<0.17	<0.014	<0.014	<0.015	<0.015	<0.013	<0.015	<0.013	<0.013	<0.013	<0.30	<0.42	<1.2	<1.2	<1.2	<6.6	<1.1	<1.2	<1.4	<1.1	<1.3	<1.3	<7.0	<1.3	<1.2	<1.3
R2	V4-R2	V4-R2-EFF	3/30/2016	--	--	<0.00050	<0.00050	<0.00050	--	--	--	<0.0025	--	<0.00050	--	--	--	--	<0.00050	<0.00050	--	--	--	--	--	--	--	--	--	--	--	--	--	--	--	--	--
R2	V5-R2	V5-R2-EFF	12/15/2015	<0.013	<0.013	<0.013	<0.013	<0.014	<0.014	<0.015	<0.013	<0.16	<0.014	<0.014	<0.014	<0.015	<0.013	<0.015	<0.013	<0.012	<0.012	<0.29	<0.42	<1.2	<1.2	<1.2	<6.6	<1.1	<1.2	<1.4	<1.1	<1.3	<1.3	<7.0	<1.3	<1.2	<1.3
R2	V5-R2	V5-R2-EFF	3/30/2016	--	--	<0.00050	<0.00050	<0.00050	--	--	--	<0.0025	--	<0.00050	--	--	--	--	<0.00050	<0.00050	--	--	--	--	--	--	--	--	--	--	--	--	--	--	--	--	--
Region 3																																					
R3	V1-R3	V1-R3-EFF	12/2/2015	<0.013	<0.013	<0.013	<0.013	<0.013	<0.013	<0.014	<0.013	<0.16	<0.014	<0.014	<0.014	<0.015	<0.013	<0.014	<0.013	<0.012	<0.012	<0.28	<0.41	<1.2	<1.2	<1.2	<6.5	<1.1	<1.1	<1.3	<1.1	<1.3	<1.3	<6.9	<1.3	<1.2	<1.3
R3	V1-R3	V1-R3-EFF	3/17/2016	--	--	<0.00050	<0.00050	<0.00050	--	--	--	<0.0025	--	<0.00050	--	--	--	--	<0.00050	<0.00050	--	--	--	--	--	--	--	--	--	--	--	--	--	--	--	--	--
R3	V2-R3	V2-R3-EFF	12/2/2015	<0.013	<0.013	<0.013	<0.013	<0.013	<0.013	<0.014	<0.013	<0.16	<0.014	<0.014	<0.014	<0.015	<0.013	<0.014	<0.013	<0.012	<0.012	<0.28	<0.41	<1.2	<1.2	<1.2	<6.5	<1.1	<1.1	<1.3	<1.1	<1.2	<1.2	<6.8	<1.3	<1.1	<1.3
R3	V2-R3	V2-R3-EFF	3/17/2016	--	--	<0.00050	<0.00050	<0.00050	--	--	--	<0.0025	--	<0.00050	--	--	--	--	<0.00050	<0.00050	--	--	--	--	--	--	--	--	--	--	--	--	--	--	--	--	--
R3	V3-R3	V3-R3-EFF	12/2/2015	<0.013	<0.013	<0.013	<0.013	<0.013	<0.013	<0.014	<0.013	<0.16	<0.014	<0.014	<0.014	<0.015	<0.013	<0.014	<0.013	<0.012	<0.012	<0.28	<0.42	<1.2	<1.2	<1.2	<6.6	<1.1	<1.2	<1.4	<1.1	<1.3	<1.3	<7.0	<1.3	<1.2	<1.3
R3	V3-R3	V3-R3-EFF	3/17/2016	--	--	<0.00050	<0.00050	<0.00050	--	--	--	<0.0025	--	<0.00050	--	--	--	--	<0.00050	<0.00050	--	--	--	--	--	--	--	--	--	--	--	--	--	--	--	--	--
R3	V4-R3	V4-R3-EFF	12/2/2015	<0.013	<0.013	<0.013	<0.013	<0.013	<0.013	<0.014	<0.013	<0.16	<0.014	<0.014	<0.014	<0.015	<0.013	<0.014	<0.013	<0.012	<0.012	<0.28	<0.42	<1.2	<1.2	<1.2	<6.6	<1.1	<1.2	<1.4	<1.1	<1.3	<1.3	<7.0	<1.3	<1.2	<1.3
R3	V4-R3	V4-R3-EFF	3/17/2016	--	--	<0.00050	<0.00050	<0.00050	--	--	--	<0.0025	--	<0.00050	--	--	--	--	<0.00050	<0.00050	--	--	--	--	--	--	--	--	--	--	--	--	--	--	--	--	--
R3	V5-R3	V5-R3-EFF	12/3/2015	<0.013	<0.013	<0.013	<0.013	<0.013	<0.013	<0.014	<0.013	<0.16	<0.014	<0.014	<0.014	<0.015	<0.013	<0.014	<0.013	<0.012	<0.012	<0.28	<0.41	<1.2	<1.2	<1.2	<6.5	<1.1	<1.1	<1.3	<1.1	<1.2	<1.2	<6.8	<1.3	<1.1	<1.3
R3	V5-R3	V5-R3-EFF	3/16/2016	--	--	<0.00050	<0.00050	<0.00050	--	--	--	<0.0025	--	<0.00050	--	--	--	--	<0.00050	<0.00050	--	--	--	--	--	--	--	--	--	--	--	--	--	--	--	--	--
Region 5																																					
R5	V1-R5	V1-R5-A	11/23/2015	<0.013	<0.013	<0.013	<0.013	<0.013	<0.013	<0.014	<0.013	<0.16	<0.014	<0.014	<0.014	<0.015	<0.013	<0.014	<0.013	<0.012	<0.012	<0.28	<0.41	<1.2	<1.2	<1.2	<6.5	<1.1	<1.1	<1.3	<1.1	<1.2	<1.2	<6.8	<1.3	<1.1	<1.3
R5	V1-R5	V1-R5-A	3/21/2016	--	--	<0.00050	<0.00050	<0.00050	--	--	--	<0.0025	--	<0.00050	--	--	--	--	<0.00050	<0.00050	--	--	--	--	--	--	--	--	--	--	--	--	--	--	--	--	--
R5	V2-R5	V2-R5-A	11/23/2015	<0.013	<0.013	<0.013	<0.013	<0.013	<0.013	<0.014	<0.013	<0.16	<0.014	<0.014	<0.014	<0.015	<0.013	<0.014	<0.013	<0.012	<0.012	<0.28	<0.41	<1.2	<1.2	<1.2	<6.5	<1.1	<1.1	<1.3	<1.1	<1.2	<1.2	<6.8	<1.3	<1.1	<1.3
R5	V2-R5	V2-R5-A	3/21/2016	--	--	<0.00050	<0.00050	<0.00050	--	--	--	<0.0025	--	<0.00050	--	--	--	--	<0.00050	<0.00050	--	--	--	--	--	--	--	--	--	--	--	--	--	--	--	--	--
R5	V3-R5	V3-R5-EFF-A	11/23/2015	<0.013	<0.013	<0.013	<0.013	<0.013	<0.013	<0.014	<0.013	<0.16	<0.014	<0.014	<0.014	<0.015	<0.013	<0.014	<0.013	<0.012	<0.012	<0.28	<0.41	<1.2	<1.2	<1.2	<6.5	<1.1	<1.1	<1.3	<1.1	<1.2	<1.2	<6.8	<1.3	<1.1	<1.3
R5	V3-R5	V3-R5-EFF-A	3/21/2016	--	--	<0.00050	<0.00050	<0.00050	--	--	--	<0.0025	--	<0.00050	--	--	--	--	<0.00050	<0.00050	--	--	--	--	--	--	--	--	--	--	--	--	--	--	--	--	--
R5	V4-R5	V4-R5-A	11/23/2015	<0.013	<0.013	<0.013	<0.013	<0.013	<0.013	<0.014	<0.013	<0.16	<0.014	<0.014	<0.014	<0.015	<0.013	<0.014	<0.013	<0.012	<0.012	<0.28	<0.41	<1.2	<1.2	<1.2	<6.5	<1.1	<1.1	<1.3	<1.1	<1.2	<1.2	<6.8	<1.3	<1.1	<1.3
R5	V4-R5	V4-R5-A	3/21/2016	--	--	<0.00050	<0.00050	<0.00050	--	--	--	<0.0025	--	<0.00050	--	--	--	--	<0.00050	<0.00050	--	--	--	--	--	--	--	--	--	--	--	--	--	--	--	--	--
R5	V5-R5	V5-R5-A	11/23/2015	<0.013	<0.013	<0.013	<0.013	<0.013	<0.013	<0.014	<0.013	<0.16	<0.014	<0.014	<0.014	<0.015	<0.013	<0.014	<0.013	<0.012	<0.012	<0.28	<0.41	<1.2	<1.2	<1.2	<6.5	<1.1	<1.1	<1.3	<1.1	<1.2	<1.2	<6.8	<1.3	<1.1	<1.3
R5	V5-R5	V5-R5-A	3/21/2016	--	--	<0.00050	<0.00050	<0.00050	--	--	--	<0.0025	--	<0.00050	--	--	--	--	<0.00050	<0.00050	--	--	--	--	--	--	--	--	--	--	--	--	--	--	--	--	--

Jeremy T. Laurin[a,b] and Allison C. Luengen[a *]
a. Environmental Sciences Department, University of San Francisco, 2130 Fulton Street, San Francisco, CA, 94117, USA
b. Environmental Management, Pacific Gas & Electric Company, 3401 Crow Canyon Road, San Ramon, CA 94583, USA
* corresponding author, email: aluengen@usfca.edu

Online Resource - Analytical Results for PG&E Utility Vault Water Samples

				SVOC	SVOC	SVOC	SVOC	SVOC	SVOC	SVOC	SVOC	SVOC	SVOC	SVOC	SVOC	SVOC	SVOC	SVOC	SVOC	SVOC	SVOC	SVOC	SVOC	SVOC
Region	Vault Location ID	Sample ID	Sample Date	4-Nitrophenol (µg/L)	Benzidine (µg/L)	Bis(2-chloroethoxy)methane (µg/L)	Bis(2-chloroethyl)ether (µg/L)	Bis(2-chloroisopropyl)ether (µg/L)	Bis(2-ethylhexyl)phthalate (µg/L)	Butyl benzyl phthalate (µg/L)	Diethyl phthalate (µg/L)	Dimethyl phthalate (µg/L)	Di-n-butyl phthalate (µg/L)	Di-n-octyl phthalate (µg/L)	Hexachlorobenzene (µg/L)	Hexachlorocyclopentadiene (µg/L)	Hexachloroethane (µg/L)	Isophorone (µg/L)	Nitrobenzene (µg/L)	N-Nitrosodimethylamine (µg/L)	N-Nitrosodi-n-propylamine (µg/L)	N-Nitrosodiphenylamine (µg/L)	Pentachlorophenol (µg/L)	Phenol (µg/L)
Water Quality Criteria (µg/L)				--	0.00054	--	1.4	--	5.9	5200	120000	2900000	12000	--	0.00077	17000	8.9	600	1900	8.1	1.4	16	pH Based	4600000
Water Quality Criteria (mg/L)				--	0.00000054	--	0.0014	--	0.0059	5.2	120	2900	12	--	0.00000077	17	0.0089	0.6	1.9	0.0081	0.0014	0.016	pH Based	4600
Minimum Level (µg/L)				5	5	5	1	2	5	10	2	2	10	10	1	5	1	1	1	5	5	1	1	1
Region 1																								
R1	V1-R1	V1-R1-EFF-A	1/7/2016	<0.77	<3.2	<1.2	<1.2	<1.6	<1.5	<1.2	<1.3	<1.3	<1.4	<1.2	<1.5	<3.4	<1.5	<1.2	<1.5	<1.6	<1.1	<1.3	<2.3	<1.0
R1	V1-R1	V1-R1-EFF-A	4/5/2016	--	--	--	--	--	--	--	--	--	--	--	<0.0018	--	--	--	--	--	--	--	--	--
R1	V2-R1	V2-R1-EFF-A	1/7/2016	<0.78	<3.2	<1.2	<1.2	<1.6	<1.5	<1.2	<1.4	<1.3	<1.4	<1.2	<1.5	<3.4	<1.5	<1.2	<1.5	<1.6	<1.2	<1.4	<2.3	<1.0
R1	V2-R1	V2-R1-EFF-A	4/5/2016	--	--	--	--	--	--	--	--	--	--	--	<0.0018	--	--	--	--	--	--	--	--	--
R1	V3-R1	V3-R1-EFF-A	1/7/2016	<0.77	<3.2	<1.2	<1.2	<1.6	<1.5	<1.2	<1.3	<1.3	<1.4	<1.2	<1.5	<3.4	<1.5	<1.2	<1.5	<1.6	<1.1	<1.3	<2.3	<1.0
R1	V3-R1	V3-R1-EFF-A	4/5/2016	--	--	--	--	--	--	--	--	--	--	--	<0.0018	--	--	--	--	--	--	--	--	--
R1	V4-R1	V4-R1-EFF-A	1/7/2016	<0.77	<3.2	<1.2	<1.2	<1.6	<1.5	<1.2	<1.3	<1.3	<1.4	<1.2	<1.5	<3.4	<1.5	<1.2	<1.5	<1.6	<1.1	<1.3	<2.3	<1.0
R1	V4-R1	V4-R1-EFF-A	4/5/2016	--	--	--	--	--	--	--	--	--	--	--	<0.0018	--	--	--	--	--	--	--	--	--
R1	V5-R1	V5-R1-EFF-A	1/11/2016	<0.77	<3.2	<1.2	<1.2	<1.6	<1.5	<1.2	<1.3	<1.3	<1.4	<1.2	<1.5	<3.4	<1.5	<1.2	<1.5	<1.6	<1.1	<1.3	<2.3	<1.0
R1	V5-R1	V5-R1-EFF-A	3/28/2016	--	--	--	--	--	--	--	--	--	--	--	<0.0018	--	--	--	--	--	--	--	--	--
Region 2																								
R2	V1-R2	V1-R2-EFF	12/1/2015	<0.78	<3.2	<1.2	<1.2	<1.6	<1.5	<1.2	<1.4	<1.3	<1.4	<1.2	<1.5	<3.4	<1.5	<1.2	<1.5	<1.6	<1.2	<1.4	<2.3	<1.0
R2	V1-R2	V1-R2-EFF	3/22/2016	--	--	--	--	--	--	--	--	--	--	--	<0.0018	--	--	--	--	--	--	--	--	--
R2	V2-R2	V2-R2-EFF	12/1/2015	<0.77	<3.1	<1.2	<1.2	<1.6	<1.5	<1.2	<1.3	<1.3	<1.4	<1.2	<1.5	<3.3	<1.5	<1.2	<1.5	<1.5	<1.1	<1.3	<2.2	<0.99
R2	V2-R2	V2-R2-EFF	3/22/2016	--	--	--	--	--	--	--	--	--	--	--	<0.0018	--	--	--	--	--	--	--	--	--
R2	V3-R2	V3-R2-EFF	12/1/2015	<0.78	<3.2	<1.2	<1.2	<1.6	<1.5	<1.2	<1.4	<1.3	<1.4	<1.2	<1.5	<3.4	<1.5	<1.2	<1.5	<1.6	<1.2	<1.4	<2.3	<1.0
R2	V3-R2	V3-R2-EFF	3/22/2016	--	--	--	--	--	--	--	--	--	--	--	<0.0018	--	--	--	--	--	--	--	--	--
R2	V4-R2	V4-R2-EFF	12/15/2015	<0.78	<3.2	<1.2	<1.2	--	<1.5	<1.2	<1.4	<1.3	<1.4	<1.2	<1.5	<3.4	<1.5	<1.2	<1.5	<1.6	<1.2	<1.4	<2.3	<1.0
R2	V4-R2	V4-R2-EFF	3/30/2016	--	--	--	--	--	--	--	--	--	--	--	<0.0018	--	--	--	--	--	--	--	--	--
R2	V5-R2	V5-R2-EFF	12/15/2015	<0.78	<3.2	<1.2	<1.2	--	<1.5	<1.2	<1.4	<1.3	<1.4	<1.2	<1.5	<3.4	<1.5	<1.2	<1.5	<1.6	<1.2	<1.4	<2.3	<1.0
R2	V5-R2	V5-R2-EFF	3/30/2016	--	--	--	--	--	--	--	--	--	--	--	<0.0018	--	--	--	--	--	--	--	--	--
Region 3																								
R3	V1-R3	V1-R3-EFF	12/2/2015	<0.77	<3.2	<1.2	<1.2	<1.6	<1.5	<1.2	<1.3	<1.3	<1.4	<1.2	<1.5	<3.4	<1.5	<1.2	<1.5	<1.6	<1.1	<1.3	<2.3	<1.0
R3	V1-R3	V1-R3-EFF	3/17/2016	--	--	--	--	--	--	--	--	--	--	--	<0.0018	--	--	--	--	--	--	--	--	--
R3	V2-R3	V2-R3-EFF	12/2/2015	<0.77	<3.1	<1.2	<1.2	<1.6	<1.5	<1.2	<1.3	<1.3	<1.4	<1.2	<1.5	<3.3	<1.5	<1.2	<1.5	<1.5	<1.1	<1.3	<2.2	<0.99
R3	V2-R3	V2-R3-EFF	3/17/2016	--	--	--	--	--	--	--	--	--	--	--	<0.0018	--	--	--	--	--	--	--	--	--
R3	V3-R3	V3-R3-EFF	12/2/2015	<0.78	<3.2	<1.2	<1.2	<1.6	<1.5	<1.2	<1.4	<1.3	<1.4	<1.2	<1.5	<3.4	<1.5	<1.2	<1.5	<1.6	<1.2	<1.4	<2.3	<1.0
R3	V3-R3	V3-R3-EFF	3/17/2016	--	--	--	--	--	--	--	--	--	--	--	<0.0018	--	--	--	--	--	--	--	--	--
R3	V4-R3	V4-R3-EFF	12/2/2015	<0.78	<3.2	<1.2	<1.2	<1.6	<1.5	<1.2	<1.4	<1.3	<1.4	<1.2	<1.5	<3.4	<1.5	<1.2	<1.5	<1.6	<1.2	<1.4	<2.3	<1.0
R3	V4-R3	V4-R3-EFF	3/17/2016	--	--	--	--	--	--	--	--	--	--	--	<0.0018	--	--	--	--	--	--	--	--	--
R3	V5-R3	V5-R3-EFF	12/3/2015	<0.77	<3.1	<1.2	<1.2	<1.6	<1.5	<1.2	<1.3	<1.3	<1.4	<1.2	<1.5	<3.3	<1.5	<1.2	<1.5	<1.5	<1.1	<1.3	<2.2	<0.99
R3	V5-R3	V5-R3-EFF	3/16/2016	--	--	--	--	--	--	--	--	--	--	--	<0.0018	--	--	--	--	--	--	--	--	--
Region 5																								
R5	V1-R5	V1-R5-A	11/23/2015	<0.77	<3.1	<1.2	<1.2	<1.6	<1.5	<1.2	<1.3	<1.3	<1.4	<1.2	<1.5	<3.3	<1.5	<1.2	<1.5	<1.5	<1.1	<1.3	<2.2	<0.99
R5	V1-R5	V1-R5-A	3/21/2016	--	--	--	--	--	--	--	--	--	--	--	<0.0018	--	--	--	--	--	--	--	--	--
R5	V2-R5	V2-R5-A	11/23/2015	<0.77	<3.1	<1.2	<1.2	<1.6	<1.5	<1.2	<1.3	<1.3	<1.4	<1.2	<1.5	<3.3	<1.5	<1.2	<1.5	<1.5	<1.1	<1.3	<2.2	<0.99
R5	V2-R5	V2-R5-A	3/21/2016	--	--	--	--	--	--	--	--	--	--	--	<0.0018	--	--	--	--	--	--	--	--	--
R5	V3-R5	V3-R5-EFF-A	11/23/2015	<0.77	<3.1	<1.2	<1.2	<1.6	<1.5	<1.2	<1.3	<1.3	<1.4	<1.2	<1.5	<3.3	<1.5	<1.2	<1.5	<1.5	<1.1	<1.3	<2.2	<0.99
R5	V3-R5	V3-R5-EFF-A	3/21/2016	--	--	--	--	--	--	--	--	--	--	--	<0.0018	--	--	--	--	--	--	--	--	--
R5	V4-R5	V4-R5-A	11/23/2015	<0.77	<3.1	<1.2	<1.2	<1.6	<1.5	<1.2	<1.3	<1.3	<1.4	<1.2	<1.5	<3.3	<1.5	<1.2	<1.5	<1.5	<1.1	<1.3	<2.2	<0.99
R5	V4-R5	V4-R5-A	3/21/2016	--	--	--	--	--	--	--	--	--	--	--	<0.0018	--	--	--	--	--	--	--	--	--
R5	V5-R5	V5-R5-A	11/23/2015	<0.77	<3.1	<1.2	<1.2	<1.6	<1.5	<1.2	<1.3	<1.3	<1.4	<1.2	<1.5	<3.3	<1.5	<1.2	<1.5	<1.5	<1.1	<1.3	<2.2	<0.99
R5	V5-R5	V5-R5-A	3/21/2016	--	--	--	--	--	--	--	--	--	--	--	<0.0018	--	--	--	--	--	--	--	--	--

				TPH	TPH	VOC	VOC	VOC	VOC	VOC	VOC	VOC	VOC	VOC	VOC	VOC	VOC	VOC	VOC
Region	Vault Location ID	Sample ID	Sample Date	TPH-Gasoline (µg/L)	TPH-Middle Distillate (C12-C21) (µg/L)	1,1,1,2-Tetrachloroethane (µg/L)	1,1,1-Trichloroethane (µg/L)	1,1,2,2-Tetrachloroethane (µg/L)	1,1,2-Trichloroethane (µg/L)	1,1,2-Trichlorotrifluoroethane (Freon 113) (µg/L)	1,1-Dichloroethane (µg/L)	1,1-Dichloroethene (µg/L)	1,1-Dichloropropene (µg/L)	1,2,3-Trichlorobenzene (µg/L)	1,2,3-Trichloropropane (µg/L)	1,2,4-Trichlorobenzene (µg/L)	1,2,4-Trimethylbenzene (µg/L)	1,2-Dibromo-3-chloropropane (µg/L)	1,2-Dibromoethane (µg/L)
Water Quality Criteria (µg/L)				--	--	--	--	11	42	--	--	3.2	--	--	--	--	--	--	--
Water Quality Criteria (mg/L)				--	--	--	--	0.011	0.042	--	--	0.0032	--	--	--	--	--	--	--
Minimum Level (µg/L)				--	--	--	0.5	0.5	0.5	--	0.5	0.5	--	--	--	1	--	--	--
Region 1																			
R1	V1-R1	V1-R1-EFF-A	1/7/2016	<48	**15 HD,J**	<0.24	<0.19	<0.22	<0.32	<0.26	<0.19	<0.20	<0.28	<0.25	<0.25	<0.25	<0.15	<2.9	<0.34
R1	V1-R1	V1-R1-EFF-A	4/5/2016	--	--	--	--	--	--	--	--	--	--	--	--	--	--	--	--
R1	V2-R1	V2-R1-EFF-A	1/7/2016	<48	**54 HD**	<0.24	<0.19	<0.22	<0.32	<0.26	<0.19	<0.20	<0.28	<0.25	<0.25	<0.25	<0.15	<2.9	<0.34
R1	V2-R1	V2-R1-EFF-A	4/5/2016	--	--	--	--	--	--	--	--	--	--	--	--	--	--	--	--
R1	V3-R1	V3-R1-EFF-A	1/7/2016	<48	<8.0	<0.24	<0.19	<0.22	<0.32	<0.26	<0.19	<0.20	<0.28	<0.25	<0.25	<0.25	<0.15	<2.9	<0.34
R1	V3-R1	V3-R1-EFF-A	4/5/2016	--	--	--	--	--	--	--	--	--	--	--	--	--	--	--	--
R1	V4-R1	V4-R1-EFF-A	1/7/2016	<48	**12 HD,J**	<0.24	<0.19	<0.22	<0.32	<0.26	<0.19	<0.20	<0.28	<0.25	<0.25	<0.25	<0.15	<2.9	<0.34
R1	V4-R1	V4-R1-EFF-A	4/5/2016	--	--	--	--	--	--	--	--	--	--	--	--	--	--	--	--
R1	V5-R1	V5-R1-EFF-A	1/11/2016	<48	**31 HD,J**	<0.24	<0.19	<0.22	<0.32	<0.26	<0.19	<0.20	<0.28	<0.25	<0.25	<0.25	<0.15	<2.9	<0.34
R1	V5-R1	V5-R1-EFF-A	3/28/2016	--	--	--	--	--	--	--	--	--	--	--	--	--	--	--	--
Region 2																			
R2	V1-R2	V1-R2-EFF	12/1/2015	<48	<8.0	<0.24	<0.19	<0.22	<0.32	<0.26	<0.19	<0.20	<0.28	<0.25	<0.25	<0.25	<0.15	<2.9	<0.34
R2	V1-R2	V1-R2-EFF	3/22/2016	--	--	--	--	--	--	--	--	--	--	--	--	--	--	--	--
R2	V2-R2	V2-R2-EFF	12/1/2015	<48	<8.0	<0.24	<0.19	<0.22	<0.32	<0.26	<0.19	<0.20	<0.28	<0.25	<0.25	<0.25	<0.15	<2.9	<0.34
R2	V2-R2	V2-R2-EFF	3/22/2016	--	--	--	--	--	--	--	--	--	--	--	--	--	--	--	--
R2	V3-R2	V3-R2-EFF	12/1/2015	<48	<8.0	<0.24	<0.19	<0.22	<0.32	<0.26	<0.19	<0.20	<0.28	<0.25	<0.25	<0.25	<0.15	<2.9	<0.34
R2	V3-R2	V3-R2-EFF	3/22/2016	--	--	--	--	--	--	--	--	--	--	--	--	--	--	--	--
R2	V4-R2	V4-R2-EFF	12/15/2015	<48	<7.7	<0.24	<0.19	<0.22	<0.32	<0.26	<0.19	<0.20	<0.28	<0.25	<0.25	<0.25	<0.15	<2.9	<0.34
R2	V4-R2	V4-R2-EFF	3/30/2016	--	--	--	--	--	--	--	--	--	--	--	--	--	--	--	--
R2	V5-R2	V5-R2-EFF	12/15/2015	<48	**10 HD,J**	<0.24	<0.19	<0.22	<0.32	<0.26	<0.19	<0.20	<0.28	<0.25	<0.25	<0.25	<0.15	<2.9	<0.34
R2	V5-R2	V5-R2-EFF	3/30/2016	--	--	--	--	--	--	--	--	--	--	--	--	--	--	--	--
Region 3																			
R3	V1-R3	V1-R3-EFF	12/2/2015	<48	<8.0	<0.24	**0.36 J**	<0.22	<0.32	<0.26	<0.19	<0.20	<0.28	<0.25	<0.25	<0.25	<0.15	<2.9	<0.34
R3	V1-R3	V1-R3-EFF	3/17/2016	--	--	--	--	--	--	--	--	--	--	--	--	--	--	--	--
R3	V2-R3	V2-R3-EFF	12/2/2015	<48	**22 HD,J**	<0.24	<0.19	<0.22	<0.32	<0.26	<0.19	<0.20	<0.28	<0.25	<0.25	<0.25	<0.15	<2.9	<0.34
R3	V2-R3	V2-R3-EFF	3/17/2016	--	--	--	--	--	--	--	--	--	--	--	--	--	--	--	--
R3	V3-R3	V3-R3-EFF	12/2/2015	<48	**14 HD,J**	<0.24	<0.19	<0.22	<0.32	<0.26	<0.19	<0.20	<0.28	<0.25	<0.25	<0.25	<0.15	<2.9	<0.34
R3	V3-R3	V3-R3-EFF	3/17/2016	--	--	--	--	--	--	--	--	--	--	--	--	--	--	--	--
R3	V4-R3	V4-R3-EFF	12/2/2015	<48	**14 HD,J**	<0.24	<0.19	<0.22	<0.32	<0.26	<0.19	<0.20	<0.28	<0.25	<0.25	<0.25	<0.15	<2.9	<0.34
R3	V4-R3	V4-R3-EFF	3/17/2016	--	--	--	--	--	--	--	--	--	--	--	--	--	--	--	--
R3	V5-R3	V5-R3-EFF	12/3/2015	<48	**11 HD,J**	<0.24	<0.19	<0.22	<0.32	<0.26	<0.19	<0.20	<0.28	<0.25	<0.25	<0.25	<0.15	<2.9	<0.34
R3	V5-R3	V5-R3-EFF	3/16/2016	--	--	--	--	--	--	--	--	--	--	--	--	--	--	--	--
Region 5																			
R5	V1-R5	V1-R5-A	11/23/2015	<48	<8.0	<0.24	<0.19	<0.22	<0.32	<0.26	<0.19	<0.20	<0.28	<0.25	<0.25	<0.25	<0.15	<2.9	<0.34
R5	V1-R5	V1-R5-A	3/21/2016	--	--	--	--	--	--	--	--	--	--	--	--	--	--	--	--
R5	V2-R5	V2-R5-A	11/23/2015	<48	<7.7	<0.24	<0.19	<0.22	<0.32	<0.26	<0.19	<0.20	<0.28	<0.25	<0.25	<0.25	<0.15	<2.9	<0.34
R5	V2-R5	V2-R5-A	3/21/2016	--	--	--	--	--	--	--	--	--	--	--	--	--	--	--	--
R5	V3-R5	V3-R5-EFF-A	11/23/2015	<48	<7.7	<0.24	<0.19	<0.22	<0.32	<0.26	<0.19	<0.20	<0.28	<0.25	<0.25	<0.25	<0.15	<2.9	<0.34
R5	V3-R5	V3-R5-EFF-A	3/21/2016	--	--	--	--	--	--	--	--	--	--	--	--	--	--	--	--
R5	V4-R5	V4-R5-A	11/23/2015	<48	<8.0	<0.24	<0.19	<0.22	<0.32	<0.26	<0.19	<0.20	<0.28	<0.25	<0.25	<0.25	<0.15	<2.9	<0.34
R5	V4-R5	V4-R5-A	3/21/2016	--	--	--	--	--	--	--	--	--	--	--	--	--	--	--	--
R5	V5-R5	V5-R5-A	11/23/2015	<48	**11 HD,J**	<0.24	<0.19	<0.22	<0.32	<0.26	<0.19	<0.20	<0.28	<0.25	<0.25	<0.25	<0.15	<2.9	<0.34
R5	V5-R5	V5-R5-A	3/21/2016	--	--	--	--	--	--	--	--	--	--	--	--	--	--	--	--

Jeremy T. Laurin[a,b] and Allison C. Luengen[a *]
a. Environmental Sciences Department, University of San Francisco, 2130 Fulton Street, San Francisco, CA, 94117, USA
b. Environmental Management, Pacific Gas & Electric Company, 3401 Crow Canyon Road, San Ramon, CA 94583, USA
* corresponding author, email: aluengen@usfca.edu

Online Resource - Analytical Results for PG&E Utility Vault Water Samples

				VOC	VOC	VOC	VOC	VOC	VOC	VOC	VOC	VOC	VOC	VOC	VOC	VOC	VOC	VOC	VOC	VOC	VOC	VOC	VOC	VOC	VOC	VOC	VOC	VOC	VOC	VOC	VOC	VOC	VOC	VOC	VOC	VOC	VOC	VOC	VOC	VOC	VOC
Region	Vault Location ID	Sample ID	Sample Date	1,2-Dichlorobenzene (µg/L)	1,2-Dichloroethane (µg/L)	1,2-Dichloropropane (µg/L)	1,3,5-Trimethylbenzene (µg/L)	1,3-Dichlorobenzene (µg/L)	1,3-Dichloropropane (µg/L)	1,4-Dichlorobenzene (µg/L)	2,2-Dichloropropane (µg/L)	2-Butanone (MEK) (µg/L)	2-Chloroethyl Vinyl Ether (µg/L)	2-Chlorotoluene (µg/L)	2-Hexanone (µg/L)	4-Chlorotoluene (µg/L)	4-Methyl-2-pentanone (MIBK) (µg/L)	Acetone (µg/L)	Acetonitrile (µg/L)	Acrolein (µg/L)	Acrylonitrile (µg/L)	Allyl Chloride (µg/L)	Benzene (µg/L)	Bromobenzene (µg/L)	Bromochloromethane (µg/L)	Bromodichloromethane (µg/L)	Bromoform (µg/L)	Bromomethane (µg/L)	Carbon Disulfide (µg/L)	Carbon Tetrachloride (µg/L)	Chlorobenzene (µg/L)	Chloroethane (µg/L)	Chloroform (µg/L)	Chloromethane (µg/L)	Chloroprene (µg/L)	cis-1,2-Dichloroethene (µg/L)	cis-1,3-Dichloropropene (µg/L)	Dibromochloromethane (µg/L)	Dibromomethane (µg/L)	Dichlorodifluoromethane (Freon 12) (µg/L)	Diisopropyl ether (µg/L)
			Water Quality Criteria (µg/L)	17000	99	39	--	2600	--	2600	--	--	--	--	--	--	--	--	--	780	0.66	--	71	--	--	46	360	4000	--	4.4	21000	--	--	--	--	--	1700	34	--	--	--
			Water Quality Criteria (mg/L)	17	0.099	0.039	--	2.6	--	2.6	--	--	--	--	--	--	--	--	--	0.78	0.00066	--	0.071	--	--	0.046	0.36	4	--	0.0044	21	--	--	--	--	--	1.7	0.034	--	--	--
			Minimum Level (µg/L)	0.5	0.5	0.5	--	0.5	--	0.5	--	--	1	--	--	--	--	--	--	2	2	--	0.5	--	--	0.5	0.5	1	--	0.5	0.5	0.5	0.5	0.5	--	--	0.5	0.5	--	--	--
Region 1																																									
R1	V1-R1	V1-R1-EFF-A	1/7/2016	<0.17	<0.18	<0.24	<0.33	<0.17	<0.24	<0.31	<0.42	<2.9	<3.1	<0.34	<2.6	<0.33	<2.7	<3.5	<2.2	<8.8	<5.2	<0.36	<0.32	<0.33	<0.38	<0.20	<0.34	<0.38	<0.44	<0.22	<0.14	<0.34	<0.22	<0.22	<0.24	<0.24	<0.18	<0.24	<0.34	<0.24	<0.24
R1	V1-R1	V1-R1-EFF-A	4/5/2016	--	--	--	--	--	--	--	--	--	--	--	--	--	--	--	--	--	<0.36	--	--	--	--	--	--	--	--	--	--	--	--	--	--	--	--	--	--	--	--
R1	V2-R1	V2-R1-EFF-A	1/7/2016	<0.17	<0.18	<0.24	<0.33	<0.17	<0.24	<0.31	<0.42	<2.9	<3.1	<0.34	<2.6	<0.33	<2.7	<3.5	<2.2	<8.8	<5.2	<0.36	<0.32	<0.33	<0.38	<0.20	<0.34	<0.38	<0.44	<0.22	<0.14	<0.34	<0.22	<0.22	<0.24	<0.24	<0.18	<0.24	<0.34	<0.24	<0.24
R1	V2-R1	V2-R1-EFF-A	4/5/2016	--	--	--	--	--	--	--	--	--	--	--	--	--	--	--	--	--	<0.36	--	--	--	--	--	--	--	--	--	--	--	--	--	--	--	--	--	--	--	--
R1	V3-R1	V3-R1-EFF-A	1/7/2016	<0.17	<0.18	<0.24	<0.33	<0.17	<0.24	<0.31	<0.42	<2.9	<3.1	<0.34	<2.6	<0.33	<2.7	<3.5	<2.2	<8.8	<5.2	<0.36	<0.32	<0.33	<0.38	<0.20	<0.34	<0.38	<0.44	<0.22	<0.14	<0.34	<0.22	<0.22	<0.24	<0.24	<0.18	<0.24	<0.34	<0.24	<0.24
R1	V3-R1	V3-R1-EFF-A	4/5/2016	--	--	--	--	--	--	--	--	--	--	--	--	--	--	--	--	--	<0.36	--	--	--	--	--	--	--	--	--	--	--	--	--	--	--	--	--	--	--	--
R1	V4-R1	V4-R1-EFF-A	1/7/2016	<0.17	<0.18	<0.24	<0.33	<0.17	<0.24	<0.31	<0.42	<2.9	<3.1	<0.34	<2.6	<0.33	<2.7	<3.5	<2.2	<8.8	<5.2	<0.36	<0.32	<0.33	<0.38	<0.20	<0.34	<0.38	<0.44	<0.22	<0.14	<0.34	<0.22	**0.40 J**	<0.24	<0.24	<0.18	<0.24	<0.34	<0.24	<0.24
R1	V4-R1	V4-R1-EFF-A	4/5/2016	--	--	--	--	--	--	--	--	--	--	--	--	--	--	--	--	--	<0.36	--	--	--	--	--	--	--	--	--	--	--	--	--	--	--	--	--	--	--	--
R1	V5-R1	V5-R1-EFF-A	1/11/2016	<0.17	<0.18	<0.24	<0.33	<0.17	<0.24	<0.31	<0.42	<2.9	<3.1	<0.34	<2.6	<0.33	<2.7	<3.5	<2.2	<8.8	<5.2	<0.36	<0.32	<0.33	<0.38	<0.20	<0.34	<0.38	<0.44	<0.22	<0.14	<0.34	<0.22	<0.22	<0.24	<0.24	<0.18	<0.24	<0.34	<0.24	<0.24
R1	V5-R1	V5-R1-EFF-A	3/28/2016	--	--	--	--	--	--	--	--	--	--	--	--	--	--	--	--	--	<0.36	--	--	--	--	--	--	--	--	--	--	--	--	--	--	--	--	--	--	--	--
Region 2																																									
R2	V1-R2	V1-R2-EFF	12/1/2015	<0.17	<0.18	<0.24	<0.33	<0.17	<0.24	<0.31	<0.42	<2.9	<3.1	<0.34	<2.6	<0.33	<2.7	<3.5	<2.2	<8.8	<5.2	<0.36	<0.32	<0.33	<0.38	<0.20	<0.34	<0.38	<0.44	<0.22	<0.14	<0.34	<0.22	<0.22	<0.24	<0.24	<0.18	<0.24	<0.34	<0.24	<0.24
R2	V1-R2	V1-R2-EFF	3/22/2016	--	--	--	--	--	--	--	--	--	--	--	--	--	--	--	--	--	<0.36	--	--	--	--	--	--	--	--	--	--	--	--	--	--	--	--	--	--	--	--
R2	V2-R2	V2-R2-EFF	12/1/2015	<0.17	<0.18	<0.24	<0.33	<0.17	<0.24	<0.31	<0.42	<2.9	<3.1	<0.34	<2.6	<0.33	<2.7	<3.5	<2.2	<8.8	<5.2	<0.36	<0.32	<0.33	<0.38	<0.20	<0.34	<0.38	<0.44	<0.22	<0.14	<0.34	<0.22	<0.22	<0.24	<0.24	<0.18	<0.24	<0.34	<0.24	<0.24
R2	V2-R2	V2-R2-EFF	3/22/2016	--	--	--	--	--	--	--	--	--	--	--	--	--	--	--	--	--	<0.36	--	--	--	--	--	--	--	--	--	--	--	--	--	--	--	--	--	--	--	--
R2	V3-R2	V3-R2-EFF	12/1/2015	<0.17	<0.18	<0.24	<0.33	<0.17	<0.24	<0.31	<0.42	<2.9	<3.1	<0.34	<2.6	<0.33	<2.7	<3.5	<2.2	<8.8	<5.2	<0.36	<0.32	<0.33	<0.38	<0.20	<0.34	<0.38	<0.44	<0.22	<0.14	<0.34	<0.22	<0.22	<0.24	<0.24	<0.18	<0.24	<0.34	<0.24	<0.24
R2	V3-R2	V3-R2-EFF	3/22/2016	--	--	--	--	--	--	--	--	--	--	--	--	--	--	--	--	--	<0.36	--	--	--	--	--	--	--	--	--	--	--	--	--	--	--	--	--	--	--	--
R2	V4-R2	V4-R2-EFF	12/15/2015	<0.17	<0.18	<0.24	<0.33	<0.17	<0.24	<0.31	<0.42	<2.9	<3.1	<0.34	<2.6	<0.33	<2.7	<3.5	<2.2	<8.8	<5.2	<0.36	<0.32	<0.33	<0.38	<0.20	<0.34	<0.38	<0.44	<0.22	<0.14	<0.34	<0.22	<0.22	<0.24	<0.24	<0.18	<0.24	<0.34	<0.24	<0.24
R2	V4-R2	V4-R2-EFF	3/30/2016	--	--	--	--	--	--	--	--	--	--	--	--	--	--	--	--	--	<0.36	--	--	--	--	--	--	--	--	--	--	--	--	--	--	--	--	--	--	--	--
R2	V5-R2	V5-R2-EFF	12/15/2015	<0.17	<0.18	<0.24	<0.33	<0.17	<0.24	<0.31	<0.42	<2.9	<3.1	<0.34	<2.6	<0.33	<2.7	<3.5	<2.2	<8.8	<5.2	<0.36	<0.32	<0.33	<0.38	<0.20	<0.34	<0.38	<0.44	<0.22	<0.14	<0.34	<0.22	<0.22	<0.24	<0.24	<0.18	<0.24	<0.34	<0.24	<0.24
R2	V5-R2	V5-R2-EFF	3/30/2016	--	--	--	--	--	--	--	--	--	--	--	--	--	--	--	--	--	<0.36	--	--	--	--	--	--	--	--	--	--	--	--	--	--	--	--	--	--	--	--
Region 3																																									
R3	V1-R3	V1-R3-EFF	12/2/2015	<0.17	<0.18	<0.24	<0.33	<0.17	<0.24	<0.31	<0.42	<2.9	<3.1	<0.34	<2.6	<0.33	<2.7	<3.5	<2.2	<8.8	<5.2	<0.36	<0.32	<0.33	<0.38	<0.20	<0.34	<0.38	<0.44	<0.22	<0.14	<0.34	<0.22	<0.22	<0.24	<0.24	<0.18	<0.24	<0.34	<0.24	<0.24
R3	V1-R3	V1-R3-EFF	3/17/2016	--	--	--	--	--	--	--	--	--	--	--	--	--	--	--	--	--	<0.36	--	--	--	--	--	--	--	--	--	--	--	--	--	--	--	--	--	--	--	--
R3	V2-R3	V2-R3-EFF	12/2/2015	<0.17	<0.18	<0.24	<0.33	<0.17	<0.24	<0.31	<0.42	<2.9	<3.1	<0.34	<2.6	<0.33	<2.7	<3.5	<2.2	<8.8	<5.2	<0.36	<0.32	<0.33	<0.38	<0.20	<0.34	<0.38	<0.44	<0.22	<0.14	<0.34	<0.22	<0.22	<0.24	<0.24	<0.18	<0.24	<0.34	<0.24	<0.24
R3	V2-R3	V2-R3-EFF	3/17/2016	--	--	--	--	--	--	--	--	--	--	--	--	--	--	--	--	--	<0.36	--	--	--	--	--	--	--	--	--	--	--	--	--	--	--	--	--	--	--	--
R3	V3-R3	V3-R3-EFF	12/2/2015	<0.17	<0.18	<0.24	<0.33	<0.17	<0.24	<0.31	<0.42	<2.9	<3.1	<0.34	<2.6	<0.33	<2.7	<3.5	<2.2	<8.8	<5.2	<0.36	<0.32	<0.33	<0.38	<0.20	<0.34	<0.38	<0.44	<0.22	<0.14	<0.34	<0.22	<0.22	<0.24	<0.24	<0.18	<0.24	<0.34	<0.24	<0.24
R3	V3-R3	V3-R3-EFF	3/17/2016	--	--	--	--	--	--	--	--	--	--	--	--	--	--	--	--	--	<0.36	--	--	--	--	--	--	--	--	--	--	--	--	--	--	--	--	--	--	--	--
R3	V4-R3	V4-R3-EFF	12/2/2015	<0.17	<0.18	<0.24	<0.33	<0.17	<0.24	<0.31	<0.42	<2.9	<3.1	<0.34	<2.6	<0.33	<2.7	<3.5	<2.2	<8.8	<5.2	<0.36	<0.32	<0.33	<0.38	<0.20	<0.34	<0.38	<0.44	<0.22	<0.14	<0.34	<0.22	<0.22	<0.24	<0.24	<0.18	<0.24	<0.34	<0.24	<0.24
R3	V4-R3	V4-R3-EFF	3/17/2016	--	--	--	--	--	--	--	--	--	--	--	--	--	--	--	--	--	<0.36	--	--	--	--	--	--	--	--	--	--	--	--	--	--	--	--	--	--	--	--
R3	V5-R3	V5-R3-EFF	12/3/2015	<0.17	<0.18	<0.24	<0.33	<0.17	<0.24	<0.31	<0.42	<2.9	<3.1	<0.34	<2.6	<0.33	<2.7	<3.5	<2.2	<8.8	<5.2	<0.36	<0.32	<0.33	<0.38	<0.20	<0.34	<0.38	<0.44	<0.22	<0.14	<0.34	<0.22	<0.22	<0.24	<0.24	<0.18	<0.24	<0.34	<0.24	<0.24
R3	V5-R3	V5-R3-EFF	3/16/2016	--	--	--	--	--	--	--	--	--	--	--	--	--	--	--	--	--	<0.36	--	--	--	--	--	--	--	--	--	--	--	--	--	--	--	--	--	--	--	--
Region 5																																									
R5	V1-R5	V1-R5-A	11/23/2015	<0.17	<0.18	<0.24	<0.33	<0.17	<0.24	<0.31	<0.42	<2.9	<3.1	<0.34	<2.6	<0.33	<2.7	<3.5	<2.2	<8.8	<5.2	<0.36	<0.32	<0.33	<0.38	<0.20	<0.34	<0.38	<0.44	<0.22	<0.14	<0.34	<0.22	<0.22	<0.24	<0.24	<0.18	<0.24	<0.34	<0.24	<0.24
R5	V1-R5	V1-R5-A	3/21/2016	--	--	--	--	--	--	--	--	--	--	--	--	--	--	--	--	--	<0.36	--	--	--	--	--	--	--	--	--	--	--	--	--	--	--	--	--	--	--	--
R5	V2-R5	V2-R5-A	11/23/2015	<0.17	<0.18	<0.24	<0.33	<0.17	<0.24	<0.31	<0.42	<2.9	<3.1	<0.34	<2.6	<0.33	<2.7	<3.5	<2.2	<8.8	<5.2	<0.36	<0.32	<0.33	<0.38	<0.20	<0.34	<0.38	<0.44	<0.22	<0.14	<0.34	**0.43 J**	<0.22	<0.24	<0.24	<0.18	**0.41 J**	<0.34	<0.24	<0.24
R5	V2-R5	V2-R5-A	3/21/2016	--	--	--	--	--	--	--	--	--	--	--	--	--	--	--	--	--	<0.36	--	--	--	--	--	--	--	--	--	--	--	--	--	--	--	--	--	--	--	--
R5	V3-R5	V3-R5-EFF-A	11/23/2015	<0.17	<0.18	<0.24	<0.33	<0.17	<0.24	<0.31	<0.42	<2.9	<3.1	<0.34	<2.6	<0.33	<2.7	<3.5	<2.2	<8.8	<5.2	<0.36	<0.32	<0.33	<0.38	<0.20	<0.34	<0.38	<0.44	<0.22	<0.14	<0.34	<0.22	<0.22	<0.24	<0.24	<0.18	<0.24	<0.34	<0.24	<0.24
R5	V3-R5	V3-R5-EFF-A	3/21/2016	--	--	--	--	--	--	--	--	--	--	--	--	--	--	--	--	--	<0.36	--	--	--	--	--	--	--	--	--	--	--	--	--	--	--	--	--	--	--	--
R5	V4-R5	V4-R5-A	11/23/2015	<0.17	<0.18	<0.24	<0.33	<0.17	<0.24	<0.31	<0.42	<2.9	<3.1	<0.34	<2.6	<0.33	<2.7	<3.5	<2.2	<8.8	<5.2	<0.36	<0.32	<0.33	<0.38	<0.20	<0.34	<0.38	<0.44	<0.22	<0.14	<0.34	<0.22	<0.22	<0.24	<0.24	<0.18	<0.24	<0.34	<0.24	<0.24
R5	V4-R5	V4-R5-A	3/21/2016	--	--	--	--	--	--	--	--	--	--	--	--	--	--	--	--	--	<0.36	--	--	--	--	--	--	--	--	--	--	--	--	--	--	--	--	--	--	--	--
R5	V5-R5	V5-R5-A	11/23/2015	<0.17	<0.18	<0.24	<0.33	<0.17	<0.24	<0.31	<0.42	<2.9	<3.1	<0.34	<2.6	<0.33	<2.7	**5.8 J**	<2.2	<8.8	<5.2	<0.36	<0.32	<0.33	<0.38	<0.20	<0.34	<0.38	<0.44	<0.22	<0.14	<0.34	<0.22	<0.22	<0.24	<0.24	<0.18	<0.24	<0.34	<0.24	<0.24
R5	V5-R5	V5-R5-A	3/21/2016	--	--	--	--	--	--	--	--	--	--	--	--	--	--	--	--	--	<0.36	--	--	--	--	--	--	--	--	--	--	--	--	--	--	--	--	--	--	--	--

Jeremy T. Laurin[a,b] and Allison C. Luengen[a *]
a. Environmental Sciences Department, University of San Francisco, 2130 Fulton Street, San Francisco, CA, 94117, USA
b. Environmental Management, Pacific Gas & Electric Company, 3401 Crow Canyon Road, San Ramon, CA 94583, USA
* corresponding author, email: aluengen@usfca.edu

Online Resource - Analytical Results for PG&E Utility Vault Water Samples

Region	Vault Location ID	Sample ID	Sample Date	VOC<br>Ethanol (µg/L)	VOC<br>Ethyl Methacrylate (µg/L)	VOC<br>Ethyl tert-butyl Ether (µg/L)	VOC<br>Ethylbenzene (µg/L)	VOC<br>Hexachlorobutadiene (µg/L)	VOC<br>Iodomethane (µg/L)	VOC<br>Isobutyl Alcohol (µg/L)	VOC<br>Isopropylbenzene (µg/L)	VOC<br>m,p-Xylene (µg/L)	VOC<br>Methacrylonitrile (µg/L)	VOC<br>Methyl methacrylate (µg/L)	VOC<br>Methyl tert-butyl Ether (MTBE) (µg/L)	VOC<br>Methylene Chloride (µg/L)	VOC<br>Naphthalene (8260) (µg/L)	VOC<br>n-Butylbenzene (µg/L)	VOC<br>n-Propylbenzene (µg/L)	VOC<br>o-Xylene (µg/L)	VOC<br>p-Isopropyl Toluene (µg/L)	VOC<br>Propionitrile (µg/L)	VOC<br>sec-Butylbenzene (µg/L)	VOC<br>Styrene (µg/L)	VOC<br>t-1,4-Dichloro-2-Butene (µg/L)	VOC<br>tert-amyl methyl ether (µg/L)	VOC<br>tert-Butyl alcohol (µg/L)	VOC<br>tert-Butylbenzene (µg/L)	VOC<br>Tetrachloroethene (µg/L)	VOC<br>Tetrahydrofuran (µg/L)	VOC<br>Toluene (µg/L)	VOC<br>trans-1,2-Dichloroethene (µg/L)	VOC<br>trans-1,3-Dichloropropene (µg/L)	VOC<br>Trichloroethene (µg/L)	VOC<br>Trichlorofluoromethane (Freon 11) (µg/L)	VOC<br>Vinyl Acetate (µg/L)	VOC<br>Vinyl Chloride (µg/L)	SVOC<br>Bis (2-chloro-1-methylethyl) ether (µg/L)
Water Quality Criteria (µg/L)				--	--	--	29000	50	--	--	--	--	--	--	--	1600	--	--	--	--	--	--	--	--	--	--	--	--	8.85	--	200000	140000	--	81	--	--	525	
Water Quality Criteria (mg/L)				--	--	--	29	0.05	--	--	--	--	--	--	--	1.6	--	--	--	--	--	--	--	--	--	--	--	--	0.00885	--	200	140	--	0.081	--	--	0.525	
Minimum Level (µg/L)				--	--	--	0.5	1	--	--	--	--	--	--	--	0.5	0.2	--	--	--	--	--	--	--	--	--	--	--	0.5	--	0.5	0.5	--	0.5	--	--	0.5	
Region 1																																						
R1	V1-R1	V1-R1-EFF-A	1/7/2016	<17	<1.4	<0.22	<0.32	<0.48	<6.8	<6.7	<0.42	<0.24	<2.5	<1.3	<0.29	<0.38	<0.41	<0.34	<0.38	<0.39	<0.14	<3.1	<0.23	<0.32	<3.0	<0.24	<4.1	<0.38	<0.22	<2.2	<0.26	<0.26	<0.35	<0.23	<0.25	<2.2	<0.27	--
R1	V1-R1	V1-R1-EFF-A	4/5/2016	--	--	--	--	--	--	--	--	--	--	--	--	--	--	--	--	--	--	--	--	--	--	--	--	--	--	--	--	--	--	--	--	--	--	--
R1	V2-R1	V2-R1-EFF-A	1/7/2016	<17	<1.4	<0.22	<0.32	<0.48	<6.8	<6.7	<0.42	<0.24	<2.5	<1.3	<0.29	<0.38	<0.41	<0.34	<0.38	<0.39	<0.14	<3.1	<0.23	<0.32	<3.0	<0.24	<4.1	<0.38	<0.22	<2.2	<0.26	<0.26	<0.35	<0.23	<0.25	<2.2	<0.27	--
R1	V2-R1	V2-R1-EFF-A	4/5/2016	--	--	--	--	--	--	--	--	--	--	--	--	--	--	--	--	--	--	--	--	--	--	--	--	--	--	--	--	--	--	--	--	--	--	--
R1	V3-R1	V3-R1-EFF-A	1/7/2016	<17	<1.4	<0.22	<0.32	<0.48	<6.8	<6.7	<0.42	<0.24	<2.5	<1.3	<0.29	<0.38	<0.41	<0.34	<0.38	<0.39	<0.14	<3.1	<0.23	<0.32	<3.0	<0.24	<4.1	<0.38	<0.22	<2.2	<0.26	<0.26	<0.35	<0.23	<0.25	<2.2	<0.27	--
R1	V3-R1	V3-R1-EFF-A	4/5/2016	--	--	--	--	--	--	--	--	--	--	--	--	--	--	--	--	--	--	--	--	--	--	--	--	--	--	--	--	--	--	--	--	--	--	--
R1	V4-R1	V4-R1-EFF-A	1/7/2016	<17	<1.4	<0.22	<0.32	<0.48	<6.8	<6.7	<0.42	<0.24	<2.5	<1.3	<0.29	<0.38	<0.41	<0.34	<0.38	<0.39	<0.14	<3.1	<0.23	<0.32	<3.0	<0.24	<4.1	<0.38	<0.22	<2.2	<0.26	<0.26	<0.35	<0.23	<0.25	<2.2	<0.27	--
R1	V4-R1	V4-R1-EFF-A	4/5/2016	--	--	--	--	--	--	--	--	--	--	--	--	--	--	--	--	--	--	--	--	--	--	--	--	--	--	--	--	--	--	--	--	--	--	--
R1	V5-R1	V5-R1-EFF-A	1/11/2016	<17	<1.4	<0.22	<0.32	<0.48	<6.8	<6.7	<0.42	<0.24	<2.5	<1.3	<0.29	<0.38	<0.41	<0.34	<0.38	<0.39	<0.14	<3.1	<0.23	<0.32	<3.0	<0.24	<4.1	<0.38	<0.22	<2.2	<0.26	<0.26	<0.35	<0.23	<0.25	<2.2	<0.27	--
R1	V5-R1	V5-R1-EFF-A	3/28/2016	--	--	--	--	--	--	--	--	--	--	--	--	--	--	--	--	--	--	--	--	--	--	--	--	--	--	--	--	--	--	--	--	--	--	--
Region 2																																						
R2	V1-R2	V1-R2-EFF	12/1/2015	<17	<1.4	<0.22	<0.32	<0.48	<6.8	<6.7	<0.42	<0.24	<2.5	<1.3	<0.29	<0.38	<0.41	<0.34	<0.38	<0.39	<0.14	<3.1	<0.23	<0.32	<3.0	<0.24	<4.1	<0.38	<0.22	<2.2	<0.26	<0.26	<0.35	<0.23	<0.25	<2.2	<0.27	--
R2	V1-R2	V1-R2-EFF	3/22/2016	--	--	--	--	--	--	--	--	--	--	--	--	--	--	--	--	--	--	--	--	--	--	--	--	--	--	--	--	--	--	--	--	--	--	--
R2	V2-R2	V2-R2-EFF	12/1/2015	<17	<1.4	<0.22	<0.32	<0.48	<6.8	<6.7	<0.42	<0.24	<2.5	<1.3	<0.29	<0.38	<0.41	<0.34	<0.38	<0.39	<0.14	<3.1	<0.23	<0.32	<3.0	<0.24	<4.1	<0.38	<0.22	<2.2	<0.26	<0.26	<0.35	<0.23	<0.25	<2.2	<0.27	--
R2	V2-R2	V2-R2-EFF	3/22/2016	--	--	--	--	--	--	--	--	--	--	--	--	--	--	--	--	--	--	--	--	--	--	--	--	--	--	--	--	--	--	--	--	--	--	--
R2	V3-R2	V3-R2-EFF	12/1/2015	<17	<1.4	<0.22	<0.32	<0.48	<6.8	<6.7	<0.42	<0.24	<2.5	<1.3	<0.29	<0.38	<0.41	<0.34	<0.38	<0.39	<0.14	<3.1	<0.23	<0.32	<3.0	<0.24	<4.1	<0.38	<0.22	<2.2	<0.26	<0.26	<0.35	<0.23	<0.25	<2.2	<0.27	--
R2	V3-R2	V3-R2-EFF	3/22/2016	--	--	--	--	--	--	--	--	--	--	--	--	--	--	--	--	--	--	--	--	--	--	--	--	--	--	--	--	--	--	--	--	--	--	--
R2	V4-R2	V4-R2-EFF	12/15/2015	<17	<1.4	<0.22	<0.32	<0.48	<6.8	<6.7	<0.42	<0.24	<2.5	<1.3	<0.29	<0.38	<0.41	<0.34	<0.38	<0.39	<0.14	<3.1	<0.23	<0.32	<3.0	<0.24	<4.1	<0.38	<0.22	<2.2	<0.26	<0.26	<0.35	<0.23	<0.25	<2.2	<0.27	<1.6
R2	V4-R2	V4-R2-EFF	3/30/2016	--	--	--	--	--	--	--	--	--	--	--	--	--	--	--	--	--	--	--	--	--	--	--	--	--	--	--	--	--	--	--	--	--	--	--
R2	V5-R2	V5-R2-EFF	12/15/2015	<17	<1.4	<0.22	<0.32	<0.48	<6.8	<6.7	<0.42	<0.24	<2.5	<1.3	<0.29	<0.38	<0.41	<0.34	<0.38	<0.39	<0.14	<3.1	<0.23	<0.32	<3.0	<0.24	<4.1	<0.38	<0.22	<2.2	<0.26	<0.26	<0.35	<0.23	<0.25	<2.2	<0.27	<1.6
R2	V5-R2	V5-R2-EFF	3/30/2016	--	--	--	--	--	--	--	--	--	--	--	--	--	--	--	--	--	--	--	--	--	--	--	--	--	--	--	--	--	--	--	--	--	--	--
Region 3																																						
R3	V1-R3	V1-R3-EFF	12/2/2015	<17	<1.4	<0.22	<0.32	<0.48	<6.8	<6.7	<0.42	<0.24	<2.5	<1.3	<0.29	<0.38	<0.41	<0.34	<0.38	<0.39	<0.14	<3.1	<0.23	<0.32	<3.0	<0.24	<4.1	<0.38	<0.22	<2.2	<0.26	<0.26	<0.35	<0.23	<0.25	<2.2	<0.27	--
R3	V1-R3	V1-R3-EFF	3/17/2016	--	--	--	--	--	--	--	--	--	--	--	--	--	--	--	--	--	--	--	--	--	--	--	--	--	--	--	--	--	--	--	--	--	--	--
R3	V2-R3	V2-R3-EFF	12/2/2015	<17	<1.4	<0.22	<0.32	<0.48	<6.8	<6.7	<0.42	<0.24	<2.5	<1.3	<0.29	<0.38	<0.41	<0.34	<0.38	<0.39	<0.14	<3.1	<0.23	<0.32	<3.0	<0.24	<4.1	<0.38	<0.22	<2.2	<0.26	<0.26	<0.35	<0.23	<0.25	<2.2	<0.27	--
R3	V2-R3	V2-R3-EFF	3/17/2016	--	--	--	--	--	--	--	--	--	--	--	--	--	--	--	--	--	--	--	--	--	--	--	--	--	--	--	--	--	--	--	--	--	--	--
R3	V3-R3	V3-R3-EFF	12/2/2015	<17	<1.4	<0.22	<0.32	<0.48	<6.8	<6.7	<0.42	<0.24	<2.5	<1.3	<0.29	<0.38	<0.41	<0.34	<0.38	<0.39	<0.14	<3.1	<0.23	<0.32	<3.0	<0.24	<4.1	<0.38	<0.22	<2.2	<0.26	<0.26	<0.35	<0.23	<0.25	<2.2	<0.27	--
R3	V3-R3	V3-R3-EFF	3/17/2016	--	--	--	--	--	--	--	--	--	--	--	--	--	--	--	--	--	--	--	--	--	--	--	--	--	--	--	--	--	--	--	--	--	--	--
R3	V4-R3	V4-R3-EFF	12/2/2015	<17	<1.4	<0.22	<0.32	<0.48	<6.8	<6.7	<0.42	<0.24	<2.5	<1.3	<0.29	<0.38	<0.41	<0.34	<0.38	<0.39	<0.14	<3.1	<0.23	<0.32	<3.0	<0.24	<4.1	<0.38	<0.22	<2.2	<0.26	<0.26	<0.35	<0.23	<0.25	<2.2	<0.27	--
R3	V4-R3	V4-R3-EFF	3/17/2016	--	--	--	--	--	--	--	--	--	--	--	--	--	--	--	--	--	--	--	--	--	--	--	--	--	--	--	--	--	--	--	--	--	--	--
R3	V5-R3	V5-R3-EFF	12/3/2015	<17	<1.4	<0.22	<0.32	<0.48	<6.8	<6.7	<0.42	<0.24	<2.5	<1.3	<0.29	<0.38	<0.41	<0.34	<0.38	<0.39	<0.14	<3.1	<0.23	<0.32	<3.0	<0.24	<4.1	<0.38	<0.22	<2.2	<0.26	<0.26	<0.35	<0.23	<0.25	<2.2	<0.27	--
R3	V5-R3	V5-R3-EFF	3/16/2016	--	--	--	--	--	--	--	--	--	--	--	--	--	--	--	--	--	--	--	--	--	--	--	--	--	--	--	--	--	--	--	--	--	--	--
Region 5																																						
R5	V1-R5	V1-R5-A	11/23/2015	<17	<1.4	<0.22	<0.32	<0.48	<6.8	<6.7	<0.42	<0.24	<2.5	<1.3	<0.29	<0.38	<0.41	<0.34	<0.38	<0.39	<0.14	<3.1	<0.23	<0.32	<3.0	<0.24	<4.1	<0.38	<0.22	<2.2	<0.26	<0.26	<0.35	<0.23	<0.25	<2.2	<0.27	--
R5	V1-R5	V1-R5-A	3/21/2016	--	--	--	--	--	--	--	--	--	--	--	--	--	--	--	--	--	--	--	--	--	--	--	--	--	--	--	--	--	--	--	--	--	--	--
R5	V2-R5	V2-R5-A	11/23/2015	<17	<1.4	<0.22	<0.32	<0.48	<6.8	<6.7	<0.42	<0.24	<2.5	<1.3	<0.29	<0.38	<0.41	<0.34	<0.38	<0.39	<0.14	<3.1	<0.23	<0.32	<3.0	<0.24	<4.1	<0.38	<0.22	<2.2	<0.26	<0.26	<0.35	<0.23	<0.25	<2.2	<0.27	--
R5	V2-R5	V2-R5-A	3/21/2016	--	--	--	--	--	--	--	--	--	--	--	--	--	--	--	--	--	--	--	--	--	--	--	--	--	--	--	--	--	--	--	--	--	--	--
R5	V3-R5	V3-R5-EFF-A	11/23/2015	<17	<1.4	<0.22	<0.32	<0.48	<6.8	<6.7	<0.42	<0.24	<2.5	<1.3	<0.29	<0.38	<0.41	<0.34	<0.38	<0.39	<0.14	<3.1	<0.23	<0.32	<3.0	<0.24	<4.1	<0.38	<0.22	<2.2	<0.26	<0.26	<0.35	<0.23	<0.25	<2.2	<0.27	--
R5	V3-R5	V3-R5-EFF-A	3/21/2016	--	--	--	--	--	--	--	--	--	--	--	--	--	--	--	--	--	--	--	--	--	--	--	--	--	--	--	--	--	--	--	--	--	--	--
R5	V4-R5	V4-R5-A	11/23/2015	<17	<1.4	<0.22	<0.32	<0.48	<6.8	<6.7	<0.42	<0.24	<2.5	<1.3	<0.29	<0.38	<0.41	<0.34	<0.38	<0.39	<0.14	<3.1	<0.23	<0.32	<3.0	<0.24	<4.1	<0.38	<0.22	<2.2	<0.26	<0.26	<0.35	<0.23	<0.25	<2.2	<0.27	--
R5	V4-R5	V4-R5-A	3/21/2016	--	--	--	--	--	--	--	--	--	--	--	--	--	--	--	--	--	--	--	--	--	--	--	--	--	--	--	--	--	--	--	--	--	--	--
R5	V5-R5	V5-R5-A	11/23/2015	<17	<1.4	<0.22	<0.32	<0.48	<6.8	<6.7	<0.42	<0.24	<2.5	<1.3	<0.29	<0.38	<0.41	<0.34	<0.38	<0.39	<0.14	<3.1	<0.23	<0.32	<3.0	<0.24	**4.8 J**	<0.38	<0.22	**6.0**	<0.26	<0.26	<0.35	<0.23	<0.25	<2.2	<0.27	--
R5	V5-R5	V5-R5-A	3/21/2016	--	--	--	--	--	--	--	--	--	--	--	--	--	--	--	--	--	--	--	--	--	--	--	--	--	--	--	--	--	--	--	--	--	--	--

Notes:
BU = Sample analyzed after holding time expired.
BV = Sample received after holding time expired.
Hardness Based = Water Quality Criteria varied based on each specific samples analytical result for Total Hardness (as CoCO3) and corresponding Hardness-based Criteria identified in Table G-4 of the General Permit.
HD = The chromatographic pattern was inconsistent with the profile of the reference fuel standard.
J = Analyte was detected at a concentration below the reporting limit and above the laboratory method detection limit. Reported value is estimated.
mg/L = milligram per liter
ng/L = nanogram/Liter
pH Based = Water Quality Criteria varied based on each specific samples analytical result for pH and corresponding Pentachlorophenol Criteria identified in Table G-5 of the General Permit.
pg/L = picogram/Liter
µg/L = microgram per liter
< = less than
-- = not applicable
Detections are **bolded**.
Exceedences of Water Quality Criteria are shaded.
